# Epidemiology and Phylogenetic Analysis of Viral Respiratory Infections in Vietnam

**DOI:** 10.3389/fmicb.2020.00833

**Published:** 2020-05-15

**Authors:** Lu Lu, Gail Robertson, Jordan Ashworth, Anh Pham Hong, Ting Shi, Alasdair Ivens, Guy Thwaites, Stephen Baker, Mark Woolhouse

**Affiliations:** ^1^Usher Institute, The University of Edinburgh, Edinburgh, United Kingdom; ^2^Statistical Consultancy Unit, School of Mathematics, The University of Edinburgh, Edinburgh, United Kingdom; ^3^Institute of Evolutionary Biology, The University of Edinburgh, Edinburgh, United Kingdom; ^4^Hospital for Tropical Diseases, Wellcome Trust Major Overseas Programme, Oxford University Clinical Research Unit, Ho Chi Minh City, Vietnam; ^5^Institute of Immunology and Infection Research, The University of Edinburgh, Edinburgh, United Kingdom; ^6^Cambridge Institute of Therapeutic Immunology and Infectious Disease (CITIID), Department of Medicine, University of Cambridge, Cambridge, United Kingdom

**Keywords:** epidemiology, viral respiratory infections, metagenomic sequencing, novel genomes, phylogeographic transmission

## Abstract

Acute respiratory infections (ARIs) impose a major public health burden on fragile healthcare systems of developing Southeast Asian countries such as Vietnam. The epidemiology, genetic diversity and transmission patterns of respiratory viral pathogens that circulate in this region are not well characterized. We used RT-PCR to screen for 14 common respiratory viruses in nasal/throat samples from 4326 ARI patients from 5 sites in Vietnam during 2012–2016. 64% of patients tested positive for viruses; 14% tested positive multiple co-infecting viruses. The most frequently detected viruses were *Respiratory syncytial virus* (RSV, 23%), *Human Rhinovirus* (HRV, 13%), *Influenza A virus* (IAV, 11%) and *Human Bocavirus* (HBoV, 7%). RSV infections peaked in July to October, were relatively more common in children <1 year and in the northernmost hospital. IAV infections peaked in December to February and were relatively more common in patients >5 years in the central region. Coinfection with IAV or RSV was associated with increased disease severity compared with patients only infected with HBoV or HRV. Over a hundred genomes belonging to 13 families and 24 genera were obtained via metagenomic sequencing, including novel viruses and viruses less commonly associated with ARIs. Phylogenetic and phylogeographic analyses further indicated that neighboring countries were the most likely source of many virus lineages causing ARIs in Vietnam and estimated the period that specific lineages have been circulating. Our study illustrates the value of applying the state-of-the-art virus diagnostic methods (multiplex RT-PCR and metagenomic sequencing) and phylodynamic analyses at a national level to generate an integrated picture of viral ARI epidemiology.

## Introduction

Acute respiratory infections are the leading cause of morbidity and mortality in young children under 5 years, accounting for over 16% of deaths worldwide, which mainly occur in Southeast Asia (Walker et al., 2013), including Vietnam, which is a densely populated country and an emerging disease hotspot (Rabaa et al., 2015). ARIs are a major public health problem in Vietnam (Tran et al., 2016), and are among the most common causes of childhood hospitalizations in certain regions (Anh et al., 2009; Anders et al., 2015). Although studies have been conducted on few respiratory pathogens such as RSV and IAV at single locations in Vietnam (Yoshida et al., 2010, 2013; Tran et al., 2013; Ho et al., 2018), no previous study has compared the clinical and molecular epidemiology of different viruses associated with ARIs across the country.

In recent years, advances in molecular diagnostic methods (such as real-time RT-PCR assays) have allowed the identification of new pathogens as causes of respiratory diseases (van de Pol et al., 2011; Jain et al., 2015b). However, there is relatively limited genome-wide sequence information on a range of recently discovered respiratory viral pathogens [e.g., HBoV and *Human metapneumovirus* (HPMV)], although such knowledge may aid studies of the circulation and spread of the viruses at both regional and global scales. Metagenomic sequencing technology and bioinformatics analyses are promising strategies for identifying pathogens in clinical and public health settings, which not only allow the characterization of different known and novel pathogens simultaneously, but also provided better understanding of the transmission dynamics of infectious diseases with the supplement of epidemiological information (Kao et al., 2014; Prachayangprecha et al., 2014).

In this study, we explored the characteristics of multiple viral pathogens co-circulating in patients with ARIs in five regions of Vietnam over a 4 year period. Specifically, we examined the differences in the relative abundance of respiratory virus infections by geographic location, patient age, and season/time, compared the severity of infections and tested for an impact of coinfections on severity. We also assessed the genetic diversity of respiratory viral pathogens in Vietnam and compared the molecular epidemiology of viruses co-circulating in the same region using full genome sequences. In addition, we used genome sequence data to look for the presence of less well-studied virus species from the virome in respiratory samples, some of which may be associated with ARIs.

## Materials and Methods

### Dataset

The Vietnam Initiative on Zoonotic InfectiONS (VIZIONS) was a multidisciplinary Vietnam-based project established to increase the understanding of the origins, risks and emergence of zoonotic infections (Rabaa et al., 2015). In this paper, we focused on samples collected from patients with acute respiratory clinical syndrome. The inclusion/exclusion criteria for respiratory patients into the VIZIONS study were: patient must have an acute respiratory tract infection as a clinical diagnosis on presentation, with fever or a history of fever in the previous 7 days and respiratory symptoms as the chief complaint; patients whose symptoms were considered not to be associated with an infectious agent, who were not resident within the province of the hospital they were attending, or who had been previously hospitalized within 6 months were excluded. Patient data used in this analysis were: admission date, age, gender, and date/outcome at discharge. Nasal and throat (NT) swabs were collected from each patient on the day of enrolment. Samples were stored at −80°C and transported to the laboratory of Oxford University Clinical Research Unit (OUCRU), HCMC, where samples were pooled for further analysis as per study protocols.

### Virus Detection

#### RT-PCR (Real Time PCR)

For all the respiratory samples collected, we tested for 14 common pathogens by RT-PCR following protocols described previously: the 4-tube real time multiplex RT-PCR was applied to screen for HMPV, *Human parainfluenza virus* (HPIV group 1–4), HBoV, EV, HRV (A, B, and C), Coronavirus (CoV), HAdV, and *Human parechovirus (HPeV*) (Jansen et al., 2011); whereas RSV (type A and B), IAV (H1 and H3) and IBV were tested for using single-target RT-PCR (Do et al., 2012; Thi Ty Hang et al., 2017). Note that the primers and probes used for HRV were specific for HRV (A, B, and C) which did not cross-react with other human enteroviruses, and vice versa.

#### Metagenomic Sequencing

Three hundred representative clinical samples were randomly picked from total samples collected in Dong Thap Province (*n* = 559) and then put through a metagenomic sequencing process which has been previously described (Rabaa et al., 2015). Assembled contigs were screened for viral origin by conducting alignments using BLASTx and BLASTn. All final viral genomes were examined for appropriate assembly based on length and the presence of the expected open reading frames. Partial genome sequences, sequences contained gaps and lower coverage areas, were removed from further phylogenetic analysis. Full genomic sequences determined in this study were deposited into GenBank; the information of genome sequences and correlated samples are given in Supplementary Table S7.

### Phylogenetic Analysis

We conducted phylogenetic analyses to further explore the evolution and transmission of current circulating respiratory viral pathogens in Vietnam using whole genome sequence data. Reference sequences for related genus were obtained from GenBank up to 16/06/2017, and aligned with sequences retrieved in this study using Muscle (Edgar, 2004). For pathogens with segmented genome (Influenza viruses), surface genes and polymerase genes were separated into gene-specific files for gene-based phylogenetic analyses. Phylogenetic trees for each viral pathogen group with the related reference sequences were first generated using RAxML (Stamatakis, 2005), employing maximum likelihood (ML) under 1000 bootstraps. The nucleotide substitution model used for all phylogenetic analyses was a general time reversible model (GTR) with a nucleotide site-specific rate heterogeneity with four rate categories and invariant sites. To determine if our sequence data exhibited temporal qualities, we performed an exploratory analysis with Path-O-Gen (available at http://tree.bio.ed.ac.uk/software/pathogen/) to measure root-to-tip divergence for ML trees.

Evolution of each major respiratory viral pathogens was examined using time-scaled Bayesian phylogenetic analysis in BEAST (version 1.8.4). Different substitution models, clock models and tree models were evaluated for each segment by using the stepping stone method (Baele et al., 2012). A general-time-reversal (GTR) model with gamma-distributed rate heterogeneity of 4 rate categories (C4) was chosen. A constant size model and a relaxed uncorrelated lognormal molecular clock model were chosen. The MCMC was run for 10^8^ steps and sampled every 10^4^ steps. Two independent runs were used in each segment to confirm the convergence and then combined. For major respiratory pathogens which have adequate numbers of Vietnamese sequences, we estimated the spatial diffusion dynamics between Vietnam and other locations (use continent and country as traits for discrete states). We considered the possible influence of unbalanced sampling and subsampled sequences from the same locations and isolation time. We used an asymmetric model and incorporated BSSVS to identify a sparse set of transmission rates that identify the statistically supported connectivity (Lemey et al., 2009; Edwards et al., 2011). We also estimated the expected number of transmissions (jumps) between Vietnam and other locations and time from a certain virus tree using Markov rewards (O’Brien et al., 2009).

### Statistical Analysis

Fisher’s exact tests were used to examine whether there was an association between types of viruses and location/gender/patient age/seasonality. Generalized linear models (GLMs) with log_e_-transformed response variables (length of stay in hospital, where day patient length of stay = 0.5 days) were used to compare length of time spent in hospital by patients infected with different viruses, patients positive and negative for virus infection, as well as patients with and without coinfections, including gender, age, and hospital site as potential confounding variables. We used an ANOVA-based model selection procedure to assess the importance of explanatory variables in explaining variation in the response variable. Each explanatory variable was tested for significance using likelihood ratio tests (LRTs) by removing each variable from the full model individually and using an LRT to compare models including and excluding that variable. To examine the effect of specific coinfection combinations on disease severity, we used a linear mixed model (with hospital sites as random effect *n* = 5) comparing Los of patients who single-infected with major viruses with patients who coinfections with other viral pathogens. All analyses were implemented in R version 3.4.4.

## Results

### Respiratory Viruses in Vietnam

**FIGURE 1:**
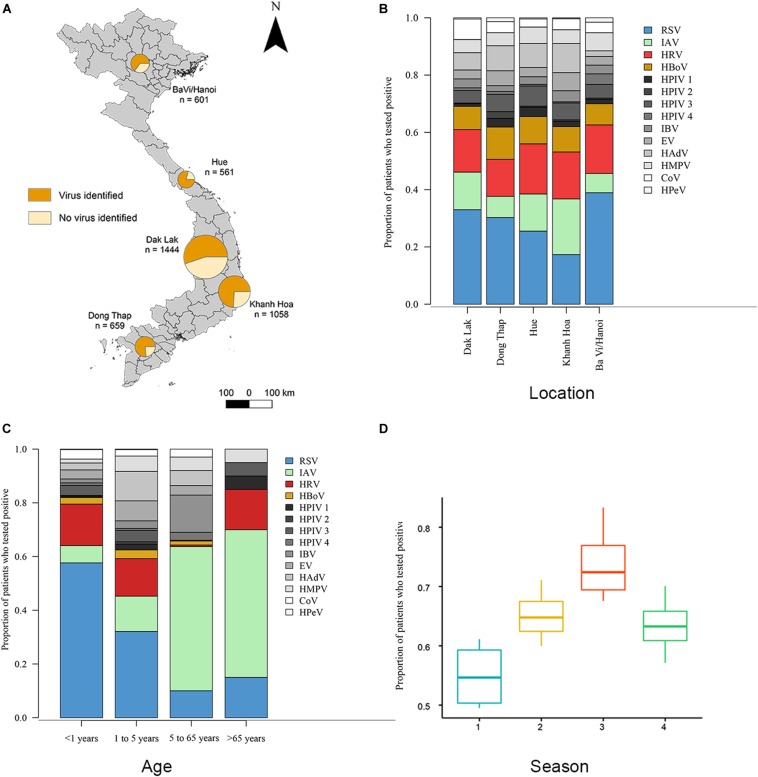
Distributions of viruses detected from ARI patients. **(A)** Proportions of patients who tested positive or negative for viral respiratory pathogens admitted to five hospital sites across Vietnam. Total *n* = 4323 (*n* = 2758 in which a virus was identified). **(B)** Proportions of 14 viral pathogens among the number of patients tested positive for at least 1 virus in each hospital site. The number of patients with viruses tested positive >300 are RSV (in blue), HRV (in red), IAV (in green) and HBoV (in yellow), other viruses are in gray. **(C)** Proportions of 14 viral pathogens among the number of patients tested positive for at least 1 virus in each admission age group. **(D)** The admit time of virus-positive ARIs grouped in four seasons from October 2012 to May 2016 (Season 1 is spring from March 1 to May 31; season 2 is summer from June 1 to August 31; season 3 is autumn from September 1 to November 30; season 4 is winter from December 1 to February 28). Full names of viruses are given in Supplementary Table S1.

This surveillance study collected nasopharyngeal and throat swabs samples from ARI patients who met pre-defined inclusion criteria from 5 hospital sites in Vietnam from November 2012 to June 2016 (Figure 1A and Supplementary Table S2). During this period, a total of 4326 respiratory patients were enrolled in the study. The male: female ratio of patients was 1:1.3. The median age of the included patients was 2 years (interquartile range, 1 to 3). The majority (*n* = 3394, 79%) were children aged 1 month to 5 years old; 844 (20%) were between 5 and 65 years; 88 (2%) were older than 65 years old. Of the 4326 enrolled patients, 4323 were tested for 14 viral pathogens using RT-PCR at hospitals in BaVi/Hanoi (*n* = 601), Hue (*n* = 561), Dak Lak (*n* = 1444), Khanh Hoa (*n* = 1058) and Dong Thap (*n* = 659) (Figure 1A). There are 2758 patients (64% of 4323) were found to be positive for at least one viral pathogen, which including 625 patients testing positive for more than one pathogen, giving a total of 3523 infections identified. The percentage of virus-positive ARI cases was higher in Dong Thap (72% of all ARIs) and Hue (75%) than in Khanh Hoa (67%), BaVi/Hanoi (59%) or Dak Lak (55%) (Figure 1A).

Overall, RSV was the most common virus identified, detected in 22.7% (*n* = 980) of tested patients, followed by HRV (12.7%, *n* = 551), IAV (10.6%, *n* = 460) and HBoV (7.3% *n* = 331). In addition, we found that the proportion of patients with ARIs who were positive for a specific viral pathogen varied significantly across hospital sites (Fisher’s exact test: *p* < 0.001) (Figure 1B). For example, among all virus positive ARI patients, the proportion of RSV in BaVi/Hanoi in the north (accounting for 0.39 of total number of virus positive patients at that site) was higher than the other viruses infections at this site; as was the proportion of IAV in Khanh Hoa in the central region (0.27 of total number of virus positive patients at that site).

Our study suggests there to be differences in the age distributions of patients infected with the 14 viruses (Kruskal–Wallis test, χ^2^_13_ = 456.2, *p* < 0.001, *n* = 3523; Supplementary Table S3). We divided patients into four age groups and compared virus proportions between them: group 1 is <1 year old (*n* = 747), group 2 is 1 to 5 years old (*n* = 2647), group 3 is 6 to 65 years old (*n* = 844), group 4 is >65 years old (*n* = 88) (Figure 1C). The percentage of virus-positive patients (those who tested positive for at least one virus) was higher in group 1 and 2 (68 and 70%) compared to group 3 and 4 (42 and 25%).

Regarding specific viruses, the percentages of RSV were significantly greater in group 1 (61% of detected viruses) compared with the other three age groups (33% in group 2, 5% in group 3 and 14% in group 4) (Fisher’s exact test: *p* < 0.001). The percentages of HRV in group 1 and 2 (20 and 20%) were significantly greater than in group 3 and 4 (12 and 9%) (Fisher’s exact test: *p* < 0.001). HBoV showed a similar pattern (9%, 11%, 3%, 5% in groups 1–4, respectively) as HRV (Fisher’s exact test: *p* < 0.001). In contrast, the percentage of IAV was higher in groups 3 and 4 (53 and 45%) and lower in groups 1 and 2 (6 and 15%) (Fisher’s exact test: *p* < 0.001).

Figure 1D and Supplementary Figure S1 show the temporal distribution for patients admitted to hospital with ARIs. The proportion of virus-positive ARIs was higher in autumn than the other seasons (Kruskal–Wallis test, χ_3_^2^ = 9.4, *p*-value = 0.02, *n* = 3523). For specific viruses, the detection of RSV peaked during May to October and IAV was most frequently detected from December to February; whereas no consistent seasonal pattern was observed for HRV and HBoV (Supplementary Figure S1). In addition, we found different subtypes of RSV and IAV circulated concurrently during three successive years (2013 to 2015). For RSV, both subtype A and subtype B were present in all 3 years: RSV-A was more commonly identified in 2013 (79% of all 146 RSV positive samples in that year) and in 2015 (67% of all 443 RSV positive samples in that year), while RSV-B was the more common subtype in 2014 (75% of all 352 RSV positive samples in that year). For IAV, the H3N2 subtype and swine-origin H1N1 (SwH1N1) subtypes were detected at similar proportions in 2013 (50% H3N2 of all 50 IAV positive cases in that year) and 2014 (53% H3N2 of all 110 IAV positive cases in that year), while H3N2 was the more commonly subtype in 2015 (65% of all 271 IAV positive cases in that year).

We used the length of stay in hospital (LoS) of patients as a proxy for disease severity as there were no fatalities in this study. The LoS (days) varied significantly between patients who tested positive for different virus species (Supplementary Table S4). For example, compared to patients who were positive for IAV (median LoS = 6.3 days), LoS for patients infected with RSV was approximately 1 day longer; while the LoS of patients who were infected with other pathogens (such as HRV and HBoV) was significantly shorter from those infected with IAV (Supplementary Table S4).

**FIGURE 2:**
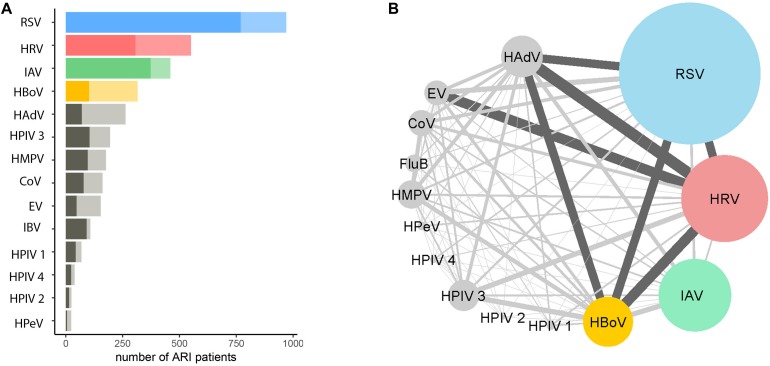
Proportions and combinations of virus co-detections among ARI patients. **(A)** The number of ARI patients infected with a single virus (darker shade) and multiple viruses. The number of patients tested positive >300 are RSV (in blue), HRV (in red), IAV (in green) and HBoV (in yellow), other viruses are in gray; shallow colors are proportions of multiple infections. **(B)** The combinations of coinfections among different viruses. The size of the nodes represented the number of patients positive for a certain virus. The width of the edges represents the number of co-detections for pairs of viruses; dark gray indicates >50. The co-detection matrix is given in Supplementary Table S5.

Among the 2758 patients who were positive for at least one viral pathogen, 2133 (77%) had a single viral pathogen and 625 (23%) had multiple viral pathogens. Overall, the length of stay in hospital for patients infected with a single virus was not significantly different from those infected with multiple viruses (Table 1A). The proportion of co-infections and virus co-infection combinations also varied among viruses (Figure 2 and Supplementary Table S5). For example, HBoV (with 67.4% cases being co-infections) most often occurred as a co-infection with other viruses, compared to HRV (with 44% cases being co-infections), RSV (with 21% cases being co-infections) and IAV (with 19% cases being co-infections). Among all RSV cases with co-infections, the most frequently co-detected viruses were HBoV (24%), HRV (24%) and HAdV (23%). Among all IAV cases with coinfections, the most frequently co-detected viruses were HBoV (34%) and HAdV (24%). Among all HRV cases with coinfections, the most frequently co-detected viruses were HAdV (28%), HBoV (25%) and *Enterovirus* (EV) (25%). Among all HBoV cases with coinfections, the most frequently co-detected viruses were HRV 29%, HAdV 24% and RSV 23%. We further tested whether co-infections with specific viruses increased the LoS but found that coinfections did not significantly affect the LoS of patients with RSV, HRV, IAV, or HBoV infections (Supplementary Table S6). However, certain virus co-infection combinations did result in longer time spent in hospital. For HBoV-infected patients, coinfection with RSV resulted in longer hospital stays (approximately 1.2 days). For IAV-infected patients, coinfection with RSV was also associated with longer stays (1.5 days). For HRV-infected patients, coinfection with RSV and HPeV were increase LoS (1.5 days). For RSV, patients who were also infected with IAV spent longer in hospital (1.5 days). All of these comparisons were made with patients who had single infections only.

**TABLE 1:** Results of normally distributed generalized linear models comparing log_e_-transformed length of stay in hospital (in days) for ARI in-patients tested using RT-PCR:

(A) Patients with multiple viruses identified (co-infections) compared to patients with a single virus identified.						

Variable	Coefficient^1^	Lower 95% confidence level	Upper 95% confidence level	LR X ^2^	df	*p*-value
Co-infection	–0.02	–0.08	0.04	0.5	1	0.46
Age	0.002	–0.0005	0.004	2.3	1	0.13
Gender	–0.03	–0.08	0.02	1.8	1	0.18
Site:	–	–	–	611.0	4	<0.001
Dak Lak	0.17	0.10	0.25	–	–	–
Hue	–0.10	–0.18	–0.02	–	–	–
Khanh Hoa	–0.52	–0.59	–0.45	–	–	–
BaVi/Hanoi	0.47	0.38	0.55	–	–	–

^1^*Results of a likelihood ratio test comparing models including and excluding pathogen (controlling for age, gender and site) are displayed, as well as estimated coefficients and their 95% confidence intervals. Total *n* = 2758 (excluding patients with no identified virus). Values displayed are given relative to patients with single infections, male patients and patients admitted in Dong Thap*.						

**(B)** Patients with no virus identified compared to patients with one or more viruses identified.						

**Variable**	**Coefficient^1^**	**Lower 95% confidence level**	**Upper 95% confidence level**	**LR X ^2^**	**df**	***p*-value**

No virus identified	–0.10	–1.41	–0.05	16.4	1	<0.001
Age	0.001	–0.0009	0.003	4.4	1	0.04
Gender	–0.05	–0.09	–0.003	4.4	1	0.04
Site:	–	–	–	640.5	4	<0.001
Dak Lak	–0.21	–0.28	–0.15	–	–	–
Hue	–0.23	–0.31	–0.15	–	–	–
Khanh Hoa	–0.66	–0.73	–0.60	–	–	–
BaVi/Hanoi	0.18	0.10	0.26	–	–	–

*^1^Results of a likelihood ratio test comparing models including and excluding pathogen (controlling for age, gender and site) are displayed, as well as estimated coefficients and their 95% confidence intervals. Total *n* = 4323. Values displayed are given relative to patients with identified viruses, male patients and patients admitted in Dong Thap.*

The same generalized linear model and likelihood ratio test was applied to virus-negative patients (*n* = 1576), who may have bacterial infections, undetected virus infections, post-viremic virus infections or non-infectious respiratory conditions. We found patients without any virus detected spent significantly less time in hospital (Table 1B). We also found that percentages of patients without any virus identified varied across hospital sites (highest in Dak Lak with 45.1% and lowest in Hue with 25.9%), and also varied across age groups (highest in older patients (aged ≥65 years).

For the most common viruses (RSV, IAV, HRV, and HBoV), we further tested whether the trends of seasonality, coinfection rate and their Los are differed among sites and age groups (Supplementary Figures S2, S3). The results were described in Supplementary Material.

### Viruses Detected by Metagenomic Sequencing

**FIGURE 3:**
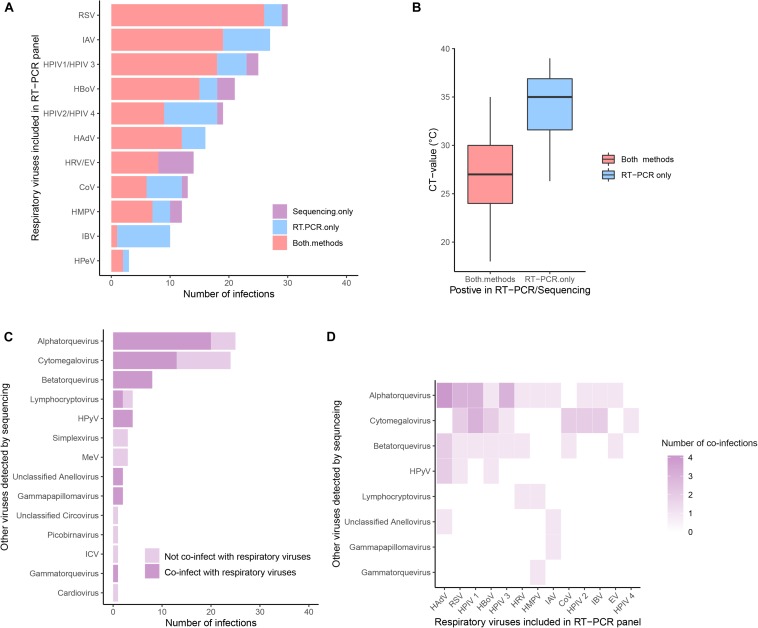
The prevalence of viruses detected by metagenomic sequencing. **(A)** Comparisons of common respiratory viruses (viruses included in RT-PCR panel, grouped by genus) detected by PCR (in blue), sequencing (in purple), or both (in red). **(B)** Comparison of CT-value in samples in which virus was detected by both methods and by PCR only. **(C)** Other viruses retrieved by sequencing. Viruses co-detected with common respiratory viruses were in a darker shade. **(D)** Heatmaps of the coinfection combinations between common respiratory viruses and other viruses.

Three hundred samples from patients admitted to hospital in southern Vietnam (Dong Thap) were further investigated using metagenomic sequencing. If multiple contigs from a sample generated hits to the same reference genome in the BLAST alignment, one representative contig was selected. In total, 218 unique full genomes/partial virus genomes (out of 1363 contigs) were resolved from 167 unique patients. These virus sequences belonged to 13 families and 24 genera (Figure 3 and Supplementary Figure S4). Around 6.3% contigs had 50–90% amino acid identity to their highest-scoring BLAST hit, mainly belonging to the family of *Anelloviridae* (Supplementary Figures S4, S6 and Supplementary Material). These may represent novel viruses.

We compared the results of metagenomic sequencing and RT-PCR (Figures 3A,B). First, we found that all 14 viral pathogens tested in the RT-PCR panel were detected in metagenomic sequencing (Figure 3A). In 123 cases the same virus was detected by both methods, in 54 cases only by RT-PCR, and in 20 cases only by metagenomics. For those samples in which a virus was only detected by RT-PCR, the Ct values (mean Ct = 33.9, SD = 3.6, *n* = 54) were significantly higher than those samples in which a virus was detected by both methods (mean Ct = 27.2, SD = 3.8, *n* = 123) (Mann-Whitney *U* Test, *p*-value < 0.001) (Figure 3B), indicating deeper sequencing might be required to detect the low levels of virus present in these samples. In addition to the “common respiratory viruses” which were tested by the RT-PCR panel (Figure 3A), metagenomic sequencing also retrieved a range of “other viruses” from 67 patients (including 27 patients not positive for any common viruses). These “other viruses” mainly belonging to *Anelloviridae* (total *n* = 36, including *Alphatorquevirus n* = 25, *Betatorquevirus n* = 8, *Gammatorquevirus n* = 1 and unclassified Anellovirus *n* = 2) and *Herpesviridae* (total *n* = 31, including *Cytomegalovirus n* = 24, *Lymphocryptovirus n* = 4, *Simplexvirus n* = 3), plus smaller numbers of HPyV, MeV, *Picobirnavirus*, *Gammapapillomavirus, Cardiovirus*, ICV, and *Circovirus* (*n* = 1 to 8) viruses (Figure 3C). Specifically, samples testing positive for ICV, *Cardiovirus*, *Picobirnavirus*, *Circovirus*, *Simplexvirus* and MeV were not found as co-infections with any common respiratory viruses (in both RT-PCR and Metagenomic sequencing). In contrast, more than half of patients who tested positive for *Alphatorquevirus* (*n* = 20), *Cytomegalovirus* (*n* = 15) and Lymphocryptovirus (*n* = 2) were co-infected with one or more of the common respiratory viruses and those who tested positive for *Betatorquevirus*, HPyV, unclassified Anellovirus, *Gammapapillomavirus* and *Gammatorquevirus* were always co-infected with common respiratory viruses. The specific co-infection combinations between common respiratory and other viruses retrieved from the same patient (*n* = 40) are shown in Figure 3D and Supplementary Figure S5.

### Evolution and Transmission of Co-circulating Viruses in ARIs in Vietnam

**FIGURE 4:**
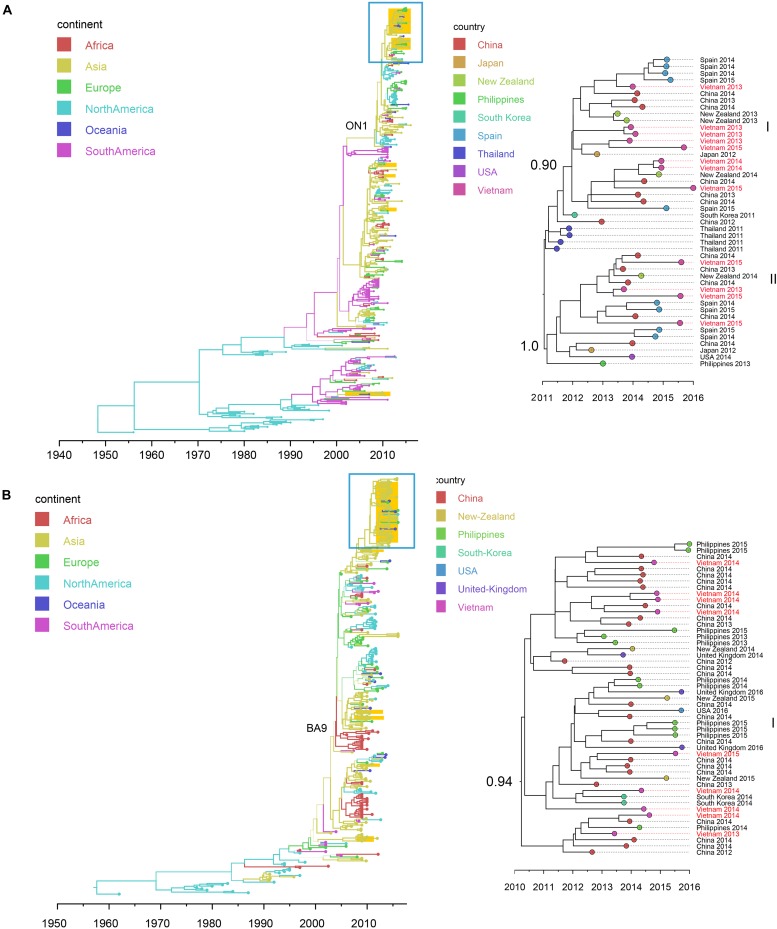
Bayesian maximum clade credibility (MCC) trees of RSV (using G gene sequences) in Vietnam and worldwide: **(A)** RSV-A (*n* = 392); **(B)** RSV-B (*n* = 314). Branch colors represent the most probable ancestral locations of each branch, inferred using a discrete trait model; sequences from Vietnam (all available) were highlighted in orange. The clades of genotype ON1 in RSV-A and genotype GA-9 in RSV-B were labeled. The clades containing Vietnamese sequences isolated in this study (within the box) are shown on the right, with tip nodes mapped to countries and isolate time (year), with Vietnamese strains highlighted in red, and labeled with subgroups and posterior support.

**FIGURE 5:**
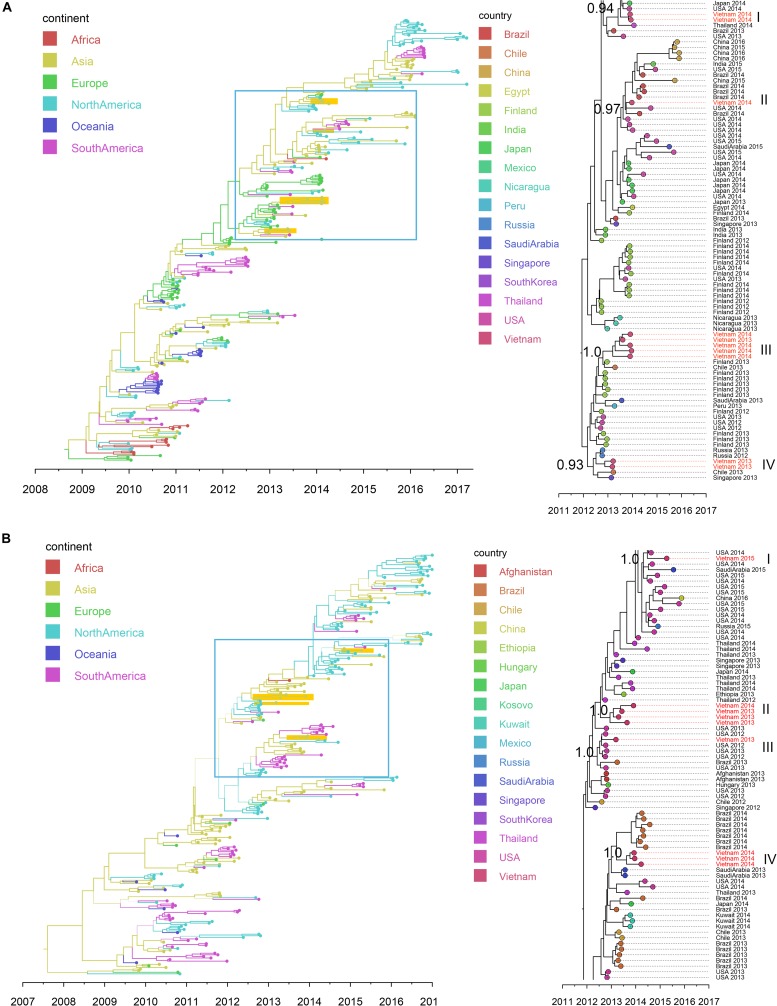
Bayesian MCC trees of IAV in Vietnam and worldwide using HA gene sequences isolated since 2009: **(A)** SwH1N1 (*n* = 296) and **(B)** H3N2 (*n* = 265). Branch colors represented the most probable ancestral locations of each branch, inferred using a discrete trait model; sequences from Vietnam (all available) were highlighted in orange. The clades containing Vietnamese sequences isolated in this study (within the box) are shown on the right, with tip nodes mapped to countries and isolate time (year), with Vietnamese strains highlighted in red, and labeled with subgroups and posterior support.

Full genome sequences (*n* = 109) were obtained, including virus species or subtypes reported for the first time in Vietnam: HAdV, HMPV, *Human coronavirus* (CoV-NL63, -229E, and -OC43), HRV, EV (type B), ICV, *Cardiovirus*, HPyV and *Anellovirus* (Supplementary Table S7). Bayesian time-scaled analyses based on genome sequences were conducted for four groups of respiratory viruses: RSV (subtypes A and B), IAV (subtypes SwH1N1 and H3N2), HBoV (subtype 1) and HPIV (group 1 to 4) (see Figures 4, 5 and Supplementary Figures S7–S9). For each viral pathogen, the estimating time to the most recent common ancestor (TMRCAs) for entire phylogenetic trees as well as individual Vietnam subgroups were estimated (Table 2) and the transmission patterns between Vietnam and other countries were further explored by using BSSVS and Markov jumps (Supplementary Table S8). For viruses that had too few sequences for spatio-temporal phylogenetic analysis (HRV, HAdV, HMPV, CoV, EV-B, MeV, ICV, HPyV, and *Anellovirus*), maximum likelihood trees were generated (Supplementary Figures S6, S10–S17). We presented the results for RSV, IAV, HRV, and HBoV in the main text below, the phylogenetic analyses of the rest viruses were given in Supplementary Material.

**TABLE 2:** Serotypes/subgroups of Vietnam sequences (RSV, IAV and HBoV) found in this study and inferred TMRCA.

Virus^1^	Vietnam subgroups			
	
	Subgroups^2^	Year of isolation	TMRCA (mean)	TMRCA (95%HPD)
RSV-A	I	2013–2015	2011.9	2011.2, 2012.4
	II	2013–2015	2011.6	2011.4, 2011.7
RSV-B	I	2013–2015	2010.6	2010.2, 2011.4
SwH1N1	I	2014	2013	2012.9, 2013.1
	II	2014	2013.4	2013.2, 2013.5
	III	2013–2014	2012.9	2012.6, 2012.9
	IV	2013	2012.4	2012.2, 2012.6
H3N2	I	2015	2014	2013.8, 2014.1
	II	2013–2014	2012.6	2012.3, 2012.6
	III	2013	2012.4	2012.3, 2012.6
	IV	2014	2013.2	2012.9, 2013.6
HBoV 1	I	2013–2014	2010.1	2007.1, 2011.3
	II	2013–2015	2006.1	2000.5, 2010.5

*^1^Viruses’ full names are given in Supplementary Table S1. ^2^Subgroups are labeled in correlated phylogenies in Figures 4, 5.*

#### Respiratory Syncytial Virus (RSV)

We retrieved 21 RSV complete genome sequences. These genomes were isolated from young children (0 to 3 years old). Twelve genomes (with nucleotide genome sequence identities between 98.6 and 99.9%) belonged to subtype A and 9 genomes (with nucleotide genome sequence identities between 99.0 and 99.7%) belonged to subtype B. There were no significant differences between the two subtypes in terms of patients’ age, gender, admission date to hospital and length of stay in hospital (Mann-Whitney *U* Test, with *p* > 0.5) (Supplementary Table S7). Bayesian time-scaled phylogenies of the G gene coding sequence and whole-genome sequences for both RSV-A and RSV-B were generated and are shown in Figure 4 and Supplementary Figure S7.

We found that all RSV-A Vietnamese isolates in this study belonged to genotype ON1 (Figure 4A and Supplementary Figure S7A). These isolates formed two well supported monophyletic clusters together with sequences within ON1 from other Asian and European countries. The mean TMRCA for the RSV-A genotype ON1 overall was 2008.1 (95%HPD 2008 to 2009), and the mean TMRCAs for both subgroups of Vietnamese strains in this study were around 2011, suggesting that these viruses had been circulating in Vietnam and adjacent areas for around 4 years (Table 2). RSV-A sequences obtained from HCMC between 2008 and 2009 belonged to a different genotype, NA1 (Do et al., 2015). In the equivalent RSV-B phylogenetic analysis, all the RSV-B Vietnamese sequences isolated in this study belonged to genotype BA9. They were in one subgroup together with sequences isolated from neighboring countries with TMRCA dating back to 2010 (Figure 4B and Table 2). The TMRCA for the RSV-B BA9 genotype overall was 2004.4 (95% HPD 2004, 2005) and for Vietnamese sequences in this study was 2010.0 (95%HPD 2009 to 2010), suggesting RSV-B viruses had been circulating in Vietnam for a similar period as RSV-A viruses (Table 2). RSV-B sequences obtained in HCMC from 2009 and 2010 also fell into the clade of genotype BA9, indicating the presence of BA9 in Vietnam at least since 2009 (Figure 3B). We found that Vietnam was more likely to be a recipient of RSV from other countries than a source (Supplementary Table S8). The most likely source populations were from China, we estimated multiple transmissions between China and Vietnam of RSV-A ON1 and RSV-B BA9 viruses between 2004 to 2016 (Supplementary Table S8).

#### Influenza A Virus (IAV)

Nineteen IAV sequences (9 H3N2 and 10 SwH1N1) with nearly full genome lengths were obtained. These IAV sequences were obtained from patients with a wide age range (0 to 35 years old) (Supplementary Table S7). Similar to RSV, we did not find significant differences between the two subtypes in terms of patients’ age, gender, admission date to hospital and length of stay in hospital (Mann-Whitney *U* Test, with *p* > 0.5). Strong temporal clustering was apparent in both SwH1N1 and H3N2 phylogenies, and sequences co-circulating in the same location in Vietnam and the same year were subdivided into multiple subgroups (four for both SwH1N1 and H3N2) (Figure 5). Each subgroup had a TMRCA around 1 year (Table 2). Specifically, the Vietnamese SwH1N1 strains isolated in 2014 fell into three different subgroups (out of the 4 in total shown in Figure 5A) and H3N2 strains from the same year fell into two subgroups (out of the 4 in total shown in Figure 5B). In addition, each subgroup was composed of strains also isolated in Vietnam and worldwide. For H1N1, three out of four subgroups of Vietnamese strains were of Asia origin, the other one was from Europe. For H3N2, 2 out of 4 subgroups were of Asia origin, the other two were from North America and Europe (Figure 5). The geographic diversity (17 different countries) and relatively small phylogenies (296 sequences for H1N1 and 265 for H3N2) results in between country transmission being estimated with low confidence (Figure 5). With currently available data, the United States was predicted as a possible source of both SwH1N1 and H3N2 in Vietnam. Other possible sources were Thailand and Finland (Supplementary Table S8).

#### Human Bocavirus (HBoV)

In total, 7 HBoV genomes were obtained, all from young children (1–2 years old). These genomes all belonged to HBoV type 1 (HBoV-1). Phylogenetic analysis of Vp1 genes showed that the co-circulating Vietnamese HBoV sequences formed two separate subgroups: three of them fell into one subgroup emerged around 2006, which were grouped together with strains isolated in HCMC in 2014 (Thanh et al., 2016), as well as with strains isolated in southern China (Zhang et al., 2015) and Thailand. The other subgroup was allocated to a distinct lineage which likely originated from a single transmission from China around 2010 (Table 2 and Supplementary Figure S8). Overall, HBoV strains were likely to be transmitted between Vietnam, China and Thailand (Supplementary Table S8).

#### Human Rhinovirus (HRV)

Ten HRV genomes were obtained in this study. Except for one isolated from a 58-year-old patient, all HRV genomes were from children under 5 years (Supplementary Table S7). The 10 Vietnamese sequences belonged to 3 genotypes (2 HRV-A, 3HRV-B, and 5 HRV-C) and 9 different HRV serotypes (A80, A82, B4, B70, B86, C11, C20, C36, and C46), indicating multiple serotypes were co-circulating in Vietnam. As these sequences were scattered over phylogenies of distinct HRV genotypes (Supplementary Figure S10) with few close related reference strains available, estimation of TMRCA and prediction of transmission patterns of the Vietnamese HRVs was not achievable.

## Discussion

Here we present the results of a large-scale, prospective study to explore the virus diversity in patients with ARIs in Vietnam. Using multiplex PCR, we found 14 respiratory viruses in patients at different proportions at 5 hospital sites during the study period. RSV was the most frequently detected virus, followed by HRV, IAV, and HBoV, although the proportions fluctuated by patient age, location and time. These findings are in good agreement with many previous studies on hospitalized ARIs (Martin et al., 2013; Jain et al., 2015a, b; Ho et al., 2018).

Consistent with previous work (Wishaupt et al., 2017), we found no differences between multiple and single virus infections in terms of disease severity. However, the proportions and combinations of viruses co-infecting patients varied substantially across studies (Scotta et al., 2016; Wishaupt et al., 2017). We found that RSV and IAV had a similar proportion of co-infections (both much lower than for HBoV and HRV). We also found that coinfection with RSV was associated with increased disease severity (length of stay in hospital) compared with patients only infected with HBoV or HRV. Similarly, patients co-infected with RSV and IAV also tended to have longer lengths of stay than those only infected with RSV or IAV. Our estimation of the percentage of RSV in Vietnam (23%) was similar to those reported among hospitalized ARI patients in several other Asia countries including China, Indonesia, Malaysia, and Thailand (ranging from 18 to 23% (Chan et al., 2002; Yoshida et al., 2010; Ohno et al., 2013; Hu et al., 2017; Thongpan et al., 2017). The percentage of IAV (11%) was similar to that in the United States and Mexico (Jain et al., 2015b). Within Vietnam, the percentage of IAV among hospitalized ARIs in Khanh Hoa Province during 2003–2005 was previously reported as 15% (Anh et al., 2009), similar to 18% during 2012–2016 in our study, but they reported a higher percentage of RSV (23%) compared with 16% here.

We found that RSV type A and B co-occurred throughout the 3 year study period. However, the proportions of these two types differed year by year (RSV A dominant in 2013 and 2015; RSV-B dominant in 2014). In North China, subgroup B was found to be dominant in the 2012/2013 season and in the 2013/2014 the subgroup of dominance shifted from B to A (Cui et al., 2015), which differs from our observations in Vietnam. Within Vietnam, the RSV-A sequences found before 2012 were mainly genotype NA1 (Do et al., 2015; Yoshihara et al., 2016). But here we found all RSV-A sequences isolated in southern Vietnam during 2012 to 2015 belonged to ON1, with an estimated TMRCA in 2011. Similarly, for RSV-B, BA9 and other two genotypes (BA10 and BA3) were present within Vietnam before 2011 (Yoshihara et al., 2016), but all RSV-B belonged to BA9 during our study period, with an estimated TMRCA of 2009. These results supported single genotype predominance (ON1 for RSV-A and BA9 for RSV-B) of RSV in Vietnam. In addition, our phylogenetic analysis showed that co-circulating lineages RSVs in Vietnam had TMRCA 4–5 years before sampling. In comparison, the IAVs isolated during each year in Vietnam were divided into multiple subgroups, consistent with reports in the literature that IAV comprises multiple clades co-circulating on a short time scale (Ghedin et al., 2005; Nelson et al., 2006).

We also identified different spatial transmission patterns for RSV and IAV in Vietnam. We found a strong phylogeographic linkage of the RSV clusters from Vietnam with sequences within Asia and frequent transmissions of RSV from China to Vietnam (especially RSV-B). In comparison, IAVs from Vietnam were likely transmitted from the sequences prevalent both within Asia and other continents (H3N2 from the United States; SwH1N1 from the United States and Europe). The results were consistent with the complex global circulation patterns of seasonal IAV (Bedford et al., 2015). For the other respiratory viruses (e.g., HBoV and HPIV) in Vietnam, inconsistent transmission patterns were observed, with introductions both from Asian countries and other continents. However, we noted their transmissions patterns could not be clearly resolved due to low numbers of sequences available.

Interestingly, metagenomic sequencing detected a range of other viruses present in nearly one-third of ARI samples. For example, measles is a potentially serious respiratory disease and outbreaks had occurred in Vietnam during 2013–2014 (Roberts, 2015). Here, we found the same genotype D8 MeV strains that dominates in southern Vietnam (Phama et al., 2014). Anelloviruses are possible sources of ARIs, supported by other studies which detected high level diverse populations of Anelloviruses from nasal secretions from ARI patients or from respiratory tract of patients with lung transplantations and lung infections (Maggi et al., 2003; Wootton et al., 2011; Young et al., 2015). One of the novel Anellovirus genomes identified in this study had high identity to a strain isolated from a child hospitalized for severe pneumonia (Galmes et al., 2013). The other viruses were found mostly co-infections, suggesting they might not be the main cause of disease. These include HPyV, *Cytomegalovirus, Lymphocryptovirus*, and *Herpes simplex virus*, often found as co-infections in the respiratory tract or lung, mainly in patients with suppressed immune systems (Graham and Snell, 1983; Wejse et al., 2001; Williams, 2014). The roles of *Cardiovirus, Circovirus, and Picorbirnavirus* in respiratory infections are still unclear. Here, we found some evidence of their association with ARIs, because the sequences detected in ARI patients here had the highest similarity to reference strains which were also isolated from respiratory samples with ARIs in adjacent Asian countries (Tsukagoshi et al., 2010; Cui et al., 2017; Krishnamurthy and Wang, 2018).

There are limitations in this study. We note that bacterial pathogens may be present in a proportion of these patients but were not included in our assays. Further studies are needed to investigate the relationship between viral infection and bacterial infection. In addition, more diverse viromes could be revealed by sequencing all ARIs from all hospital sites, rather than a single location in Dong Thap.

Overall, our study is the most up-to-date and large-scale study describing the clinical and molecular epidemiology of viral ARIs across Vietnam. We highlighted that RSV and IAV are the two leading pathogens regarding their high prevalence and disease severity of ARIs in Vietnam. Multiple genotypes and subgroups of RSV and IAV were found co-circulating with frequent inter-country transmission and local persistence. In addition, our knowledge of virus diversity in ARIs was extended by using metagenomic sequencing. By combining genome sequences with epidemiological information, our study provided better understanding of the presence and transmissions of the full range viral pathogens associated with respiratory diseases in this population. These findings provide useful information to better guide healthcare systems in viral respiratory infection control and monitor in Vietnam and worldwide.

## Data Availability Statement

The datasets generated during the current study are available in this published article (and its Supplementary Material) and the genome sequences obtained have been submitted in Genbank with Accession Number in Supplementary Table S7.

## Ethics Statement

The studies involving human participants were reviewed and approved by the Oxford Tropical Research Ethics Committee (OxTREC) (No. 157-12) in the United Kingdom. Written informed consent to participate in this study was provided by the participants’ legal guardian/next of kin.

## Author Contributions

LL and MW designed the study. GR and LL performed the statistical analyses. JA, AI, and LL performed the metagenomics analyses. LL performed the phylogenetic analyses and wrote the manuscript. All authors reviewed, commented on and approved the manuscript.

## Conflict of Interest

The authors declare that the research was conducted in the absence of any commercial or financial relationships that could be construed as a potential conflict of interest.
