# Supplementary material for: Epidemiology and Phylogenetic Analysis of Viral Respiratory Infections in Vietnam
Source: Front Microbiol. 2020 May 15;11:833. doi: 10.3389/fmicb.2020.00833 (PMC7242649; doi:10.3389/fmicb.2020.00833)
Supplement: Supplementary file 1 [file Data_Sheet_1.pdf]

Supplementary Materials

This file includes:

Comparisons of virus infection among different sites and/or age groups

Phylogenetic analysis of respiratory virus genomes from ARI patients in Vietnam

Supplementary References

Tables S1 to S8

Figures S1 to S17

Comparisons of virus infection among different sites and/or age groups

We examined whether number of patients testing positive for the most common viruses (RSV, IAV, HRV and HBoV) varied among sites in terms of trends of seasonality (Figure S2). We used a negative binomial generalised linear model with number of patients testing positive as the response variable and a three-way interaction between site, season and virus type with associated main effects as explanatory variables. We found the three-way interaction to be significant (Likelihood Ratio Test: $X^2_{35} = 54.7$, $p = 0.018$, $n = 435$) which shows that trends in different viral infections varied across seasons in different sites. For example, in comparison with patients in BaVi/Hanoi in autumn, fewer patients tested positive for HBoV relative to other viruses in other seasons and sites. We repeated with analysis to examine whether number of patients with coinfections varies among seasons and whether seasonal patterns are similar among regions, using a negative binomial distributed GLM including an interaction between season and hospital site. We found the interaction to be significant (Likelihood Ratio Test: $X^2_{12} = 54.6$, $p<0.001$, $n = 435$) suggesting that numbers of patients with coinfections had a seasonal pattern but this pattern varied among hospital sites. These differences in trends may be driven by climate and geography of different sites of Vietnam, or differences in seasonal viral outbreaks across the country.

Similarly, we found the rate of coinfections varied among age groups and hospital sites (Likelihood ratio test: $X^2_{11} = 21.2$, $p = 0.03$, $n = 68$) (Figure S3). Patients within age group 1-5 had a higher number of coinfections than the other groups, and this number was higher in Dong Thap than the other sites.

In addition, we tested whether disease severity (Los) varied among age groups and sites in three aspects. Firstly, in the comparison made between single infections and coinfection, we found no significant interaction between hospital site and coinfection in regard to length of time spent in hospital (Likelihood Ratio Test: $X^2_4 = 5.6$, $p = 0.23$, $n = 2758$) while there was significant interaction among age and coinfection (Likelihood Ratio Test: $X^2_1 = 5.3$, $p = 0.02$, $n = 2758$). Specifically, the length of time spent in hospital for patients with single infections increased faster with age than those with coinfections. Secondly, in the comparison made between virus infection and no virus infection, we found that Los of patients who had no identified virus varied significantly among sites. For example, patients in Dong Thap with no identified virus spent longer in hospital than those admitted to other sites. Finally, when comparing the

infections of different viruses, we found that for patients infected with different viruses, Los were varied among sites and as patient age increased, Los also increased, although this trend varied depending on type of virus patient was infected with.

Phylogenetic analysis of respiratory virus genomes from ARI patients in Vietnam

Anellovirus

Five full genomes belonging to *Anelloviriae* were retrieved in metagenomic sequencing and phylogenetic analysis were further applied (Table S7 and Figure S6). Four of them (vzttmv1 to vzttmv4) are potentially new species belonging to the genus *Betatorquevirus* (Torque teno mini virus, TTMV) and one genome (vzttmv5) is an unclassified Anellovirus according to the definition used by the ICTV, i.e. 35% ORF1 gene divergence being the species demarcation and 56% being the genus demarcation [1]. Specifically, vzttmv1 is divergent from any known TTMV species (TTMV 1-12), with ORF1 nucleotide sequence divergence >=51.4%. It is most closely related to strain JX134044 (with 32.6% nt divergence), which is also an unclassified TTMV species isolated from a child hospitalised for severe pneumonia in France [2]. Virus vzttmv2 has 37.8% nt divergence from the most closely related reference sequence of species TTMV-1; vzttmv3 has 63.1% nt divergence to species TTMV-12; and vzttmv4 has 41.7% nt divergence to species TTMV -5. Vzttmv5 (Genbank no.MK212032) is divergent from any current recognized *Anellovirus* genus, with ORF1 nucleotide sequence divergence >=56.6%. It has a maximum amino acid sequence identity of 39% with Gorilla anellovirus KT027940.1 (unclassified Anelloviridae). This novel anellovirus strain was 2919nt long, encoding two putative open reading frames (665aa ORF1 and 111aa ORF2) (Figure S6 B) .

Human parainfluenza virus (HPIV)

We obtained 18 HPIV (5 HPIV 1, 3 HPIV 2, 8 HPIV3 and 2 HPIV 4) genomes and compared the molecular epidemiology of HPIV 1 to 4 (Figure S9A-D). The majority of the Vietnamese HPIV genomes were isolated from children <5 years, except for one HPIV 1 from a 76-year-old patient. For HPIV 1, the Vietnamese isolates were separated into two subgroups; two sequences (isolated in 2014) in one subclade were closely related to previously reported sequences isolated from HCMC during 2009-2010 [3]; while three sequences (isolated in 2014-2015) fell into other subclades with strains found in the US at the same time period, For HPIV

2, two sequences grouped together with previously isolated HPIV 2 in Vietnam in one clade while the other one sequence fell into a distinct clade; Eight HPIV 3 genomes isolated in this study were separated into 3 different subgroups: one subgroup fell into the lineage composed of current circulated strains from Europe; the other two subgroups were close related to reference sequences main from Vietnam (HCMC) and China. For HPIV 4, one genome belong to HPIV 4a and grouped with previously isolated HPIV 4 in Vietnam while the other fell into 4b lineage. The discrete trait analysis also suggested both local persistence and cross-country spread of HPIVs (Table S8): e.g., strong support for multiple transmissions were found from other Asian countries (China and Japan) into Vietnam for HPIV 3 and HPIV 4; One subgroup of HPIV 1 strains may come from the USA; while there was weakly supported transmission of HPIV 2 from Japan to Vietnam.

Human adenovirus (HAdV)

Multiple genotypes of HAdVs (n=9) were isolated from 1-5 year old children in southern Vietnam (Table S7). Two genomes isolated between 2013 and 2014 belonged to separate types within Adv-B (Adv-B3 and Adv-B7) (Figure S11A). Seven genomes isolated between 2014 to 2015 belonged to Adv-C: five of which clustered in Adv-C1 with high identity (97.6-99.9% nt identity); the other 2 Adv-C sequences fell into clades of Adv-C2 and Adv-C5, respectively (Figure S11 B).

Human metapneumovirus (HMPV)

There were 17 (2.6%) HMPV-positive samples in Dong Thap and 5 whole genomes were resolved from 1-5 year old children. Two closely related HMPV genotype A genomes were identified (both isolated in Oct 2013), which were similar to a strain KM361520 isolated in Thailand in Jul 2013 with 99.1-99.2% nt identity; three HMPV genotype B (isolated in Aug 2014- Dec 2014) were separated into two subgroups, with vzhmpvb1 closely related to isolates in US in 2015 while vzhmpvb2 and vzhmpv3 were grouped together and were distant from the other sequences in the same subgroups (Figure S12).

Enterovirus (EV)

Two EV- B genomes were isolated from young children (1 year and 2 year old) in April 2014 and May 2015 respectively. They fell into the clusters of two different serotypes: one belonging to serotype E11 and the other to serotype E3 (Figure S13).

Coronavirus (CoV)

We obtained 4 full genomes of CoV from respiratory samples. Two of them belonged to *Human coronavirus NL63* (vzcorona-a1 isolated from a 65-year-old patient in Aug 2014 and vzcorona-a2 isolated from an infant in Aug 2015), they fell into two separate subgroups; Vzcorona-a3 belongs to *Human coronavirus 229E* which was isolated from 1-year-old child in Oct 2013 in Vietnam (Figure S14). We also obtained a single Human coronavirus OC43 genome from a 1-year-old child in April 2015. The full genome ML tree showed that the coronaviruses were closely related to strains isolated in China and the USA in the same period of time (Figure S14).

Measles morbillivirus (MeV)

We found 3 MeV genomes in ARI patients, together with 2 more MeV genomes isolated from enteric patients in the same hospital site under the same suvelliance study [4]. These sample were negative for the 14 respiratory viruses in RT-PCR panel. These highly similar genomes (99.7-99.8% nt identity) were isolated from children age 0-1 during May to August in 2014 in Dong Thap. These sequences belonged to genotype D8, which was the dominant genotype for the outbreak in 2014 in HCMC [5]. The MeVs identified in Dong Thap and HCMC had the same unique amino acid pattern in 3 different sites (R442, S451 and G452) in the N protein as isolates. In addition, the genomes in Dong Thap were highly similar to (with 99.8% nt identity) a strain isolated in 2013 epidemic in Germany [6] (Figure S15), indicating same virus of D8 genotype may dominant in southern Vietnam and wide spread world-wide during 2013-2014.

Influenza C virus (ICV)

The only genome of ICV isolated in this study was from a 2-year-old patient. This sample was negative for the 14 respiratory viruses in RT-PCR panel. Phylogenetic trees of eight gene segments showed the Vietnamese strain found in study belongs to one Kanagawa/76 antigenic group [7], with sequences isolated mainly from East Asia during the study period [8] (Figure S16).

Human polyomavirus (HPyV)

We found 4 full-length polyomavirus genomes from 1-2-year-old children. Three of them were co-infections with at least one of the common respiratory viruses, HBoV, RSV or HAdV

(Figure 3D). Phylogenetic analysis showed one genome belonged to species HPyV-3, the other three grouped together fell into the cluster for species HPyV-4. For both species, the most closely related reference genomes to our isolates are from hospitalized children at the same time period in China. The HPyV-3 strain was closely related to a strain from a child with gastroenteritis; while the HPyV-4 viruses were closely related to a strain isolated from children with acute respiratory tract infection [9], with 99.8-99.9% nt identity (Figure S17).

Cardiovirus

The *Cardiovirus* genome obtained belonged to *Saffold virus* type 3 (SAFV-3). The 3-year-old Vietnamese patient who infected was negative for the 14 respiratory viruses in RT-PCR (Table S7). The closest related sequence (AB983594, with 96.4% nt identity over the whole genome) was acquired from a patient with upper respiratory tract inflammation in 2008 in Japan [10].

Others

IBV, HPeV, Cytomegalovirus, Lymphocryptovirus, Herpes simplex virus, Picorbirnavirus and Circovirus were represented only by partial contigs. We found that the IBV partial genomes have the highest identity (98.3-98.4%) to a strain isolated from a 9-year-old in Japan in 2014 (B/Isahaya/13I004/2014), which belongs to the Victoria lineage; and the HPeV contigs belonged to HPeV type 1 and type 4, respectively. They were closely related to strains circulated in adjacent Asian countries during the study period [11,12] with amino acid identity 93.3% and 99.4%. For Cytomegalovirus, all the cases all belong to Human betaherpesvirus 5 and have an aa identity 97.8-100% to known existing sequences in Genbank. Similarly, all Lymphocryptovirus cases possessed 99.3-100% aa identity to known Human gammaherpesvirus 4 and all Herpes simplex virus cases belong to Human alphaherpesvirus 1 with aa identity 98.7-99.5%. The contig belong to *Circoviridae* is related to an unclassified Circovirus Human PoSCV5-like circular virus which isolated from upper respiratory sample in China (only with 76.7% aa identity). Picorbirnavirus contig had 98% aa identity to novel genome sequence isolated from a respiratory swab isolate in Cambodia[13]. In addition, we identified one partial contig (553 nt) belonging to Circoviridae that is related to an unclassified Circovirus Human PoSCV5-like circular virus which isolated from upper respiratory sample in China, though with only 76.7% aa identity to its Cap protein [14].

Supplementary Reference

1. King AMQ AM, Carstens EB, Lefkowitz EJ Virus taxonomy. Elsevier,San Diego. 2012:331–341.
2. Galmes J, Li Y, Rajoharison A, et al. Potential implication of new torque teno mini viruses in parapneumonic empyema in children. Eur Respir J. 2013 Aug;42(2):470-9.
3. Linster M, Do LAH, Minh NNQ, et al. Clinical and Molecular Epidemiology of Human Parainfluenza Viruses 1-4 in Children from Viet Nam. Sci Rep. 2018 May 1;8.
4. Oude Munnink BB, Phan MV, Kellam P, et al. Complete Genome Characterization of Two Wild-Type Measles Viruses from Vietnamese Infants during the 2014 Outbreak. Genome Announc. 2016 Apr 14;4(2).
5. Phama VH, Nguyet DH, Mai KNH, et al. Measles Epidemics Among Children in Vietnam: Genomic Characterization of Virus Responsible for Measles Outbreak in Ho Chi Minh City, 2014. Ebiomedicine. 2014 Dec;1(2-3):133-140.
6. Sparrer KM, Krebs S, Jager G, et al. Complete Genome Sequence of a Wild-Type Measles Virus Isolated during the Spring 2013 Epidemic in Germany. Genome Announc. 2014 Apr 17;2(2).
7. Matsuzaki Y, Sugawara K, Mizuta K, et al. Antigenic and genetic characterization of influenza C viruses which caused two outbreaks in Yamagata City, Japan, in 1996 and 1998. J Clin Microbiol. 2002 Feb;40(2):422-429.
8. Matsuzaki Y, Sugawara K, Abiko C, et al. Epidemiological information regarding the periodic epidemics of influenza C virus in Japan (1996-2013) and the seroprevalence of antibodies to different antigenic groups. J Clin Virol. 2014 Sep;61(1):87-93.
9. Zhu T, Lu QB, Zhang SY, et al. Molecular epidemiology of WU polyomavirus in hospitalized children with acute respiratory tract infection in China. Future Microbiol. 2017 May;12:481-489.
10. Tsukagoshi H, Masuda Y, Mizutani T, et al. Sequencing and phylogenetic analyses of Saffold cardiovirus (SAFV) genotype 3 isolates from children with upper respiratory infection in Gunma, Japan. Jpn J Infect Dis. 2010 Sep;63(5):378-80.
11. Hibino A, Massaad E, Kondo H, et al. Neuraminidase inhibitor susceptibility and evolutionary analysis of human influenza B isolates from three Asian countries during 2012-2015. Infect Genet Evol. 2018 Aug;62:27-33.
12. Gong YN, Chen GW, Yang SL, et al. A Next-Generation Sequencing Data Analysis Pipeline for Detecting Unknown Pathogens from Mixed Clinical Samples and Revealing Their Genetic Diversity. PLoS One. 2016;11(3):e0151495.
13. Krishnamurthy SR, Wang D. Extensive conservation of prokaryotic ribosomal binding sites in known and novel picobirnaviruses. Virology. 2018 Mar;516:108-114.
14. Cui LB, Wu BY, Zhu XJ, et al. Identification and genetic characterization of a novel circular single-stranded DNA virus in a human upper respiratory tract sample. Arch Virol. 2017 Nov;162(11):3305-3312.

Supplementary Tables and Figures

Supplementary tables

Table S1 Abbreviations in this study

Full name	Abbreviation
Human adenovirus	HAdV
Human bocavirus	HBoV
Coronavirus	CoV
Enterovirus	EV
Influenza A virus	IAV
Swine-origin 2009 H1N1 Influenza A virus	SwH1N1
H3N2 Influenza A virus	H3N2
Influenza B virus	IBV
Influenza C virus	ICV
Human metapneumovirus	HMPV
Human parainfluenza virus 1	HPIV 1
Human parainfluenza virus 2	HPIV 2
Human parainfluenza virus 3	HPIV 3
Human parainfluenza virus 4	HPIV 4
Human parechovirus	HPeV
Human rhinovirus	HRV
Respiratory syncytial virus	RSV
Measles morbillivirus	MeV
Human polyomavirus	HPyV
Human parechovirus	HPeV
Ho Chi Minh City	HCMC
The length of stay in hospital	LoS
Standard Deviation	SD
Standard Error	SE
Estimating time to the most recent common ancestor	TMRCAs

Table S2 Hospitals, geographic and seasonal information for each site

Hospitals	Site	Region	Seasonality
Dong Thap General Hospital	Dong Thap Province	South	dry and hot from November to April, warm and wet between May and October with the highest rainfall in June to August.
Dak Lak General Hospital	Dak Lak Province	Central	dry and hot between January and August; with high rainfall in September to November.
Khanh Hoa General Hospital	Khanh Hoa Province		
Hue Central Hospital	Thua Thien Hue Province		
National Hospital for Tropical Diseases (NHTD), Ba Vi District Hospital	Ha Noi/Ba Vi	North	hot and humid with high rainfall from May to October; November to April is cooler and dry; December and January can be cold in the far north.

Table S3. Results of a negative binomial generalised linear model comparing age (in years) among patients who tested positive for different pathogens using RT-PCR.

**Variable**[1]	**Coefficient**	**Lower 95% confidence level**	**Upper 95% confidence level**	**LR X^2**	**df**	**p-value**
Pathogen:	-	-	-	1119.7.	13	<0.001
HAdV	-1.62	-1.81	-1.43	-	-	-
HBoV	-1.48	-1.76	-1.18	-	-	-
CoV	-1.14	-1.43	-0.84	-	-	-
EV	-1.60	-1.81	-1.39	-	-	-
IBV	-0.07	-0.31	0.18	-	-	-
HMPV	-1.14	-1.36	-0.91	-	-	-
HPIV 1	-1.46	-1.81	-1.07	-	-	-
HPIV 2	-1.30	-1.81	-0.72	-	-	-
HPIV 3	-0.80	-1.04	-0.56	-	-	-
HPIV 4	-1.97	-2.52	-1.37	-	-	-
HPeV	-2.11	-3.25	-0.78	-	-	-
HRV	-1.17	-1.33	-1.01	-	-	-
RSV	-1.94	-2.08	-1.81	-	-	-

[1]Values displayed for pathogen are given relative to patients with IAV. Results of a likelihood ratio test comparing models including and excluding pathogen is displayed, as well as estimated coefficients and their 95% confidence intervals. Total n = 3523.

Table S4. Results of a normally-distributed generalised linear model comparing log_e-transformed Los in hospital (in days) for ARI in-patients who were tested positive for different pathogens using RT-PCR.

**Variable**	**Coefficient[2]**	**Lower 95% confidence level**	**Upper 95% confidence level**	**LR X^2**	**df**	**p-value**
Pathogen[1]:	-	-	-	44.4	13	<0.001
HAdV	-0.12	-0.21	-0.03	-	-	-
HBoV	0.01	-0.15	0.13	-	-	-
CoV	0.03	-0.18	0.12	-	-	-
EV	-0.12	-0.22	-0.01	-	-	-
IBV	-0.05	-0.18	0.07	-	-	-
HMPV	0.12	0.003	0.23	-	-	-
HPIV 1	0.03	-0.15	0.20	-	-	-
HPIV 2	-0.27	-0.53	-0.002	-	-	-
HPIV 3	-0.04	-0.15	0.09	-	-	-
HPIV 4	-0.01	-0.27	0.25	-	-	-
HPeV	-0.08	-0.61	0.44	-	-	-
HRV	-0.001	-0.08	0.08	-	-	-
RSV	0.08	0.01	0.15	-	-	-

[1]Viruses' full names are given in Table S1.

[2] Results of a likelihood ratio test comparing models including and excluding pathogen are displayed, as well as estimated coefficients and their 95% confidence intervals. Total n=3523, which represents the total number of distinct infections because patients in this analysis may have more than one pathogen. Values displayed for each pathogen are relative to values for IAV.

Table S5 Number of specific virus co-detection combinations among ARI patients (as shown in Figure 2).

	HBo V	HPe V	Co V	HPIV 4	HPIV 3	HPIV 2	HPIV 1	HMP V	HR V	HAd V	E V	RS V	IB V	IA V
HBoV														
HPeV	3													
CoV	13	3												
HPIV 4	3	0	0											
HPIV 3	30	1	8	1										
HPIV 2	2	0	2	2	1									
HPIV 1	8	0	5	0	0	0								
HMPV	23	1	23	1	8	1	0							
HRV	61	8	17	5	29	0	8	11						
HAdV	51	4	15	3	18	1	7	20	69					
EV	16	1	1	1	10	1	1	6	62	19				
RSV	50	2	19	6	10	1	1	10	51	48	30			
IBV	6	0	1	0	1	0	1	0	1	4	2	4		
IAV	29	3	6	1	9	1	1	8	8	21	4	14	0	

Table S6 Results of a linear mixed model (LMM) comparing $\log_e$-transformed Los in hospital (in days) of patients single-infected with major viruses (RSV, IAV, HRV and HBoV) with patients coinfected with other viral pathogens.

**Variable[1]**	**Coefficient**	**SE**	**t-value**	**p-value**
**Relative to values for single infection in RSV-infected patients (Total n=980)**				
Age	0.01	0.006	2.81	0.005
Gender	-0.05	0.03	-1.67	0.09
HAdV	-0.03	0.07	-0.35	0.72
HBoV	0.09	0.07	1.26	0.21
CoV	0.18	0.11	1.61	0.11
EV	0.003	0.09	0.03	0.97
IAV	0.31	0.13	2.44	0.01
IBV	-0.07	0.24	-0.32	0.75
HMPV	0.12	0.15	0.8	0.42
HPIV 1	-0.31	0.47	-0.67	0.51
HPIV 2	0.22	0.47	0.46	0.65
HPIV 3	0.23	0.15	1.49	0.14
HPIV 4	-0.29	0.2	-1.49	0.14
HPeV	-0.43	0.33	-1.28	0.2
HRV	-0.03	0.07	-0.48	0.63
**Relative to values for single infection in IAV-infected patients (Total n=460)**				
Age	0.001	0.001	0.36	0.72
Gender	-0.1	0.05	-1.78	0.08
HAdV	0.07	0.13	0.5	0.62
HBoV	-0.04	0.12	-0.32	0.75
CoV	0.42	0.24	1.74	0.08
EV	0.08	0.3	0.25	0.8
HMPV	-0.44	0.22	-2.02	0.04
HPIV 3	0.12	0.2	0.63	0.53
HPeV	0.55	0.34	1.59	0.11
HRV	0.34	0.22	1.57	0.12
RSV	0.34	0.16	2.09	0.04
**Relative to values for single infection in HRV-infected patients (Total n=551)**				
Age	-0.002	0.002	-0.93	0.35
Gender	-0.01	0.05	-0.11	0.91
HAdV	-0.07	0.07	-0.98	0.33
HBoV	-0.04	0.07	-0.49	0.63
CoV	-0.05	0.13	-0.36	0.72
EV	-0.12	0.07	-1.59	0.11
IAV	0.32	0.19	1.65	0.1
HPIV 1	-0.27	0.19	-1.41	0.16
HPIV 3	0.005	0.1	0.05	0.96
HPIV 4	-0.14	0.25	-0.58	0.56
HPeV	0.42	0.19	2.16	0.03
RSV	0.1	0.08	1.18	0.04
**Relative to values for single infection in HBoV-infected patients (Total n=331)**				

Age	-0.02	0.02	-3.52	4.98E-04
Gender	-0.05	0.06	-0.84	0.4
HAdV	-0.1	0.09	-1.14	0.25
CoV	-0.06	0.16	-0.4	0.69
EV	-0.33	0.14	-2.3	0.02
IAV	0.02	0.11	0.18	0.85
IBV	-0.06	0.22	-0.28	0.78
HMPV	0.07	0.12	0.57	0.57
HPIV 1	-0.3	0.2	-1.47	0.14
HPIV 2	-0.12	0.39	-0.31	0.76
HPIV 3	0.15	0.1	1.43	0.16
HPIV 4	-0.18	0.32	-0.56	0.58
HPeV	-0.05	0.32	-0.15	0.88
HRV	-0.01	0.08	-0.08	0.94
RSV	0.22	0.09	2.46	0.01

[1]Viruses' full names are given in Table S1. Results of likelihood ratio tests comparing models including and excluding pathogen (controlling for age, gender and site as random effect) are displayed, as well as estimated coefficients and standard errors. For each pathogen, total n represents the total number of distinct coinfection in patients infected with such pathogen; values displayed for each pathogen are relative to values for the single-infection listed.

[2] Exclude coinfections involving IBV, HPIV 1, 2 and 4 as there were 1 or 0 cases

[3] Exclude coinfections involving IBV, HMPV and HPIV 2 as there were 1 or 0 cases

Table S7 Genome sequences of respiratory viruses retrieved in this study.

Species	Serotypes/groups	Acession.No.	Strain.name	Age	Gender	Los (D)	Date of Collection
HAdV B	type 3	MH828478	vzhadvb1	1	M	7	12/15/13
HAdV B	type 7	MH828479	vzhadvb2	2	M	5	11/6/14
HAdV C	type 1	MH828484	vzhadvc1-5	1	M	8	3/10/14
HAdV C	type 2	MH828485	vzhadvc2	4	F	5	3/12/14
HAdV C	type 1	MH828480	vzhadvc1-1	2	M	2	3/12/14
HAdV C	type 1	MH828481	vzhadvc1-2	2	M	6	7/24/14
HAdV C	type 1	MH828482	vzhadvc1-3	2	F	9	3/17/15
HAdV C	type 1	MH828483	vzhadvc1-4	2	F	4	3/29/15
HAdV C	type 5	MH828486	vzhadvc5	3	M	5	4/17/15
HBoV	type 1	MH828524	vzbov1	1	M	9	1/8/13
HBoV	type 1	MH828525	vzbov2	2	F	6	2/18/14
HBoV	type 1	MH828526	vzbov3	1	F	4	4/16/15
HBoV	type 1	MH828527	vzbov4	2	M	6	4/16/13
HBoV	type 1	MH828528	vzbov5	1	F	7	12/12/13
HBoV	type 1	MH828529	vzbov6	2	M	7	4/10/14
HBoV	type 1	MH828530	vzbov7	2	M	5	11/6/14
HCoV NL63		MH828487	vzcorona-a1	65	M	21	8/26/14
HCoV NL63		MH828488	vzcorona-a2	0	M	1	8/18/15
HCoV 229E		MH828489	vzcorona-a3	2	F	9	11/22/13
HCoV OC43		MH828490	vzcorona-b1	1	F	3	4/17/15
HRV B	B04	MH828512	vzhrvb1	1	M	5	11/12/14
HRV B	B70	MH828513	vzhrvb2	58	F	1	12/18/13
HRV B	B86	MH828514	vzhrvb3	1	F	6	5/31/13
HRV A	A82	MH828515	vzhrva1	1	M	4	3/20/14
HRV A	A80	MH828516	vzhrva2	2	F	5	1/28/15
HRV C	C11	MH828521	vzhrvc5	1	M	5	4/9/13
HRV C	C46	MH828519	vzhrvc3	4	F	4	4/25/13
HRV C	C20	MH828517	vzhrvc1	2	F	5	4/16/14
HRV C	C36	MH828520	vzhrvc4	1	F	6	6/17/14
HRV C	C46	MH828518	vzhrvc2	0	M	8	9/16/15
EV B	E3	MH828522	vzecho1	2	F	10	5/7/15
EV B	E11	MH828523	vzecho2	1	M	4	4/16/14
IAV	H1N1	MH828531-MH828538	A/Vietnam/1582/2014	2	M	23	3/9/14
IAV	H1N1	MH828539-MH828546	A/Vietnam/1544/2014	31	F	6	2/17/14
IAV	H1N1	MH828547-MH828554	A/Vietnam/1542/2014	35	M	5	2/17/14
IAV	H1N1	MH828555-MH828562	A/Vietnam/1127/2013	1	M	7	6/13/13
IAV	H1N1	MH828563-MH828570	A/Vietnam/2548/2014	1	M	6	2/19/14
IAV	H1N1	MH828571-MH828578	A/Vietnam/1492/2014	2	F	8	2/25/14
IAV	H1N1	MH828579-MH828586	A/Vietnam/1123/2013	1	M	9	6/10/13

IAV	H1N1	MH828587-MH828594	A/Vietnam/1118/2013	0	M	12	5/29/13
IAV	H1N1	MH828595-MH828602	A/Vietnam/1056/2014	19	F	6	2/28/14
IAV	H1N1	MH828603-MH828610	A/Vietnam/1545/2014	15	F	7	2/17/14
IAV	H3N2	MH828611-MH828618	A/Vietnam/1556/2015	4	F	3	6/30/15
IAV	H3N2	MH828619-MH828626	A/Vietnam/1194/2013	2	F	5	11/6/13
IAV	H3N2	MH828627-MH828634	A/Vietnam/1134/2013	1	M	5	7/10/13
IAV	H3N2	MH828635-MH828642	A/Vietnam/1122/2013	1	M	11	5/31/13
IAV	H3N2	MH828643-MH828650	A/Vietnam/3262/2014	3	F	6	2/18/14
IAV	H3N2	MH828651-MH828658	A/Vietnam/272/2013	1	M	6	8/25/13
IAV	H3N2	MH828659-MH828666	A/Vietnam/1309/2014	3	M	7	5/27/14
IAV	H3N2	MH828667-MH828673	A/Vietnam/1319/2014	2	M	5	6/12/14
IAV	H3N2	MH828675-MH828682	A/Vietnam/1315/2014	3	M	7	6/2/14
ICV	Kanagawa/76 antigenic group	MH828688 - MH828694	C/Vietnam/01/2014	2	M	2	4/16/14
HPMV	HMPV-A	MH828684	vzhmpva2	1	F	6	10/2/13
HPMV	HMPV-A	MH828683	vzhmpva1	2	M	6	10/24/13
HPMV	HMPV-B	MH828687	vzhmpvb3	1	F	7	8/14/14
HPMV	HMPV-B	MH828685	vzhmpvb1	3	F	4	11/27/14
HPMV	HMPV-B	MH828686	vzhmpvb2	1	M	15	12/18/14
HPIV 3		MH82869	vzhpiv31	1	M	5	12/17/14
HPIV 3		MH82870	vzhpiv32	1	M	7	1/10/14
HPIV 3		MH82871	vzhpiv33	2	M	5	2/7/14
HPIV 3		MH82872	vzhpiv34	1	M	5	1/23/14
HPIV 3		MH82873	vzhpiv35	1	F	4	1/15/15
HPIV 3		MH82874	vzhpiv36	1	M	13	6/17/15
HPIV 3		MH82875	vzhpiv37	3	F	5	3/13/15
HPIV 3		MH82876	vzhpiv38	2	M	7	12/22/14
HPIV 1		MH82877	vzhpiv11	3	F	7	12/8/14
HPIV 1		MH82878	vzhpiv12	76	M	21	1/13/15
HPIV 1		MH82879	vzhpiv13	1	F	7	3/31/15
HPIV 1		MH82880	vzhpiv14	2	F	6	10/16/14
HPIV 1		MH82881	vzhpiv15	1	M	7	12/1/14
HPIV 4	4b	MH82882	vzhpiv41	1	M	3	3/6/15
HPIV 4	4a	MH82883	vzhpiv42	0	M	5	6/3/15
HPIV 2		MH82884	vzhpiv21	3	F	5	5/3/14
HPIV 2		MH82885	vzhpiv22	1	M	4	5/12/14
HPIV 2		MH82886	vzhpiv23	3	M	4	9/25/15
HPyV 3		MK049348	vzki1	1	F	4	4/16/15
HPyV 4		MK049349	vzwu1	2	F	4	10/20/14
HPyV 4		MK049350	vzwu2	2	M	5	11/6/14
HPyV 4		MK049351	vzwu3	2	F	4	3/29/15
RSVA		MH828491	RSVA/Vietnam/vzrsva1/2013	0	M	8	12/10/13

RSVA		MH828492	RSVA/Vietnam/vzrsva2/2013	3	F	8	10/24/13
RSVA		MH828493	RSVA/Vietnam/vzrsva3/2013	3	F	8	11/7/13
RSVA		MH828494	RSVA/Vietnam/vzrsva4/2015	3	F	5	10/2/15
RSVA		MH828495	RSVA/Vietnam/vzrsva5/2014	1	M	7	7/28/14
RSVA		MH828496	RSVA/Vietnam/vzrsva6/2014	1	M	8	7/28/14
RSVA		MH828497	RSVA/Vietnam/vzrsva7/2015	0	M	9	9/23/15
RSVA		MH828498	RSVA/Vietnam/vzrsva8/2015	1	M	5	10/9/15
RSVA		MH828499	RSVA/Vietnam/vzrsva9/2015	0	M	6	9/29/15
RSVA		MH828500	RSVA/Vietnam/vzrsva10/2013	1	M	8	11/12/13
RSVA		MH828501	RSVA/Vietnam/vzrsva11/2013	0	M	5	10/16/13
RSVA		MH828502	RSVA/Vietnam/vzrsva12/2015	2	M	5	8/12/15
RSVB		MH828503	RSVB/Vietnam/vzrsvb1/2014	1	M	3	8/5/14
RSVB		MH828504	RSVB/Vietnam/vzrsvb2/2014	1	F	9	8/20/14
RSVB		MH828505	RSVB/Vietnam/vzrsvb3/2014	3	M	4	9/19/14
RSVB		MH828506	RSVB/Vietnam/vzrsvb4/2014	1	M	5	11/28/14
RSVB		MH828507	RSVB/Vietnam/vzrsvb5/2013	1	M	3	9/17/13
RSVB		MH828508	RSVB/Vietnam/vzrsvb6/2014	2	F	7	9/8/14
RSVB		MH828509	RSVB/Vietnam/vzrsvb7/2014	0	F	7	9/22/14
RSVB		MH828510	RSVB/Vietnam/vzrsvb8/2014	2	M	4	9/18/14
RSVB		MH828511	RSVB/Vietnam/vzrsvb9/2015	0	M	8	10/21/15
MeV	D8	MK142914	MVs/DongThap.VNM/05.14[D8]	1	F	9	5/3/14
MeV	D8	MK142915	MVs/DongThap.VNM/06.14/2[D8]	0	M	6	6/7/14
MeV	D8	MK142916	MVs/DongThap.VNM/07.14[D8]	1	F	4	7/14/14
Unclassified Anellovirus		MK139485	vzttmv1	1	M	4	3/14/14
Unclassified Anellovirus		MK212029	vzttmv2	1	M	9	2/19/14
Unclassified Anellovirus		MK212030	vzttmv3	2	M	5	11/6/14
Unclassified Anellovirus		MK212031	vzttmv4	1	F	4	5/18/15
Unclassified Anellovirus		MK212032	vzttmv5	2	F	4	3/7/14
Cardiovirus B	Saffold virus type 3	MK139484	vzsafv3	3	F	5	1/8/14

Table S8 Inferred number of virus transmissions between Vietnam and other countries

Virus[1]	Transmission	Number of Markov jumps[2]		
		median	95% HPD interval	BF support
RSV-A (ON1)	China->Vietnam	3	(1, 6)	>100
RSV-B (BA9)	China->Vietnam	13	(10, 16)	>100
H1N1 (Sw2009)	Finland->Vietnam	2	(1, 3)	8
	USA->Vietnam	2	(1, 3)	>100
H3N2	Thailand->Vietnam	1	(0, 2)	5
	USA->Vietnam	3	(1, 4)	>100
HboV-1	China->Vietnam	1	(0, 2)	69
	Thailand->Vietnam	1	(0, 1)	4
	Vietnam->China	1	(0, 1)	8
HPIV 1	USA->Vietnam	1	(0, 2)	13
HPIV 2	Japan->Vietnam	1	(0, 2)	4
HPIV 3	China->Vietnam	2	(1, 3)	>100
HPIV 4	Japan->Vietnam	3	(1, 4)	>100

[1]Viruses' full names are given in Table S1.
[2]Numbers of transitions were estimated using a Markov jumps analysis combined with the correlated Bayes Factor (BF) supports using BSSVS. Transitions with BF>3 are shown.

Supplementary figures

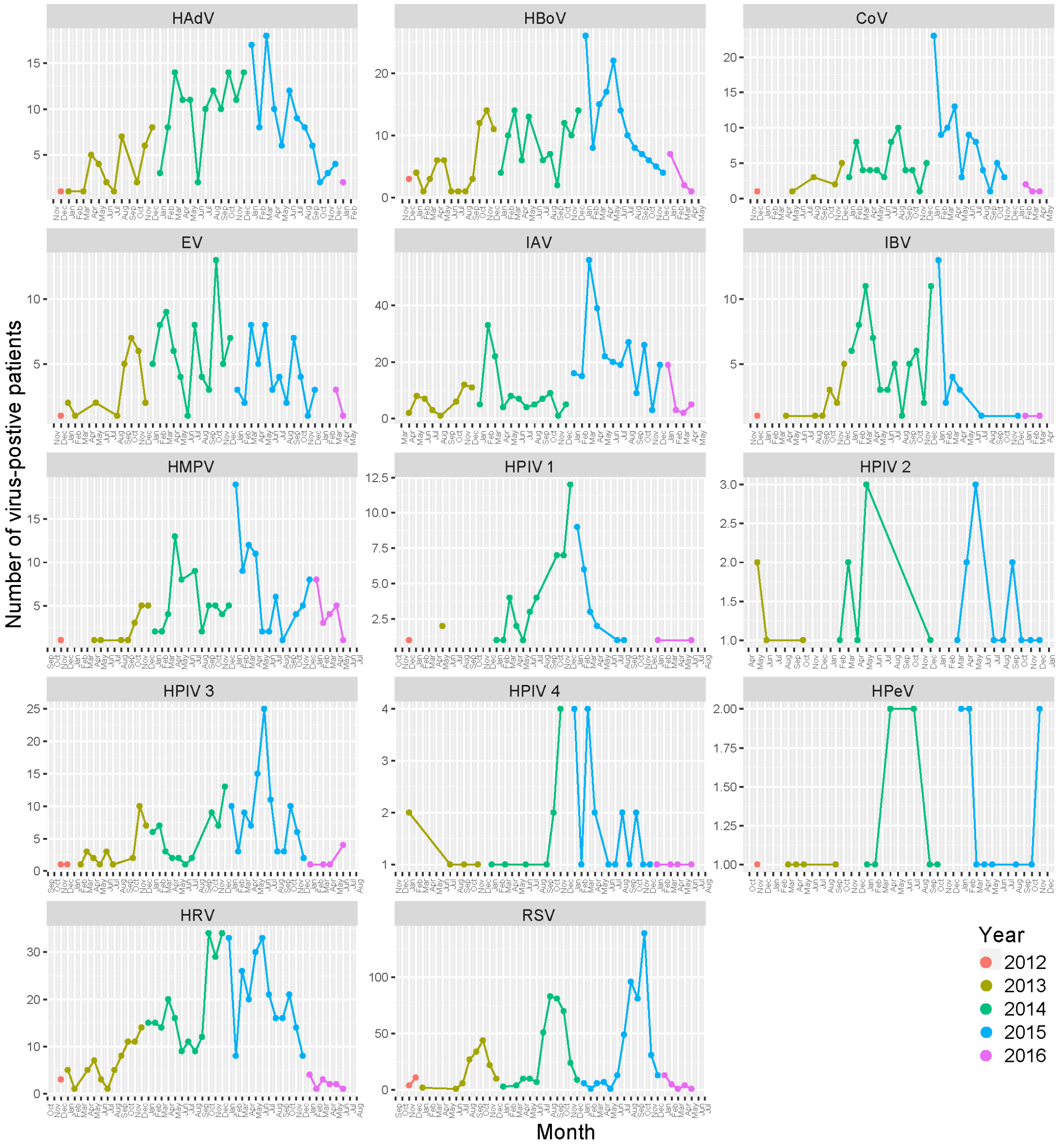

Figure S1 Monthly distribution of different viruses RT-PCR detected in ARI patients admitted to hospital from October 2012 to June 2016.

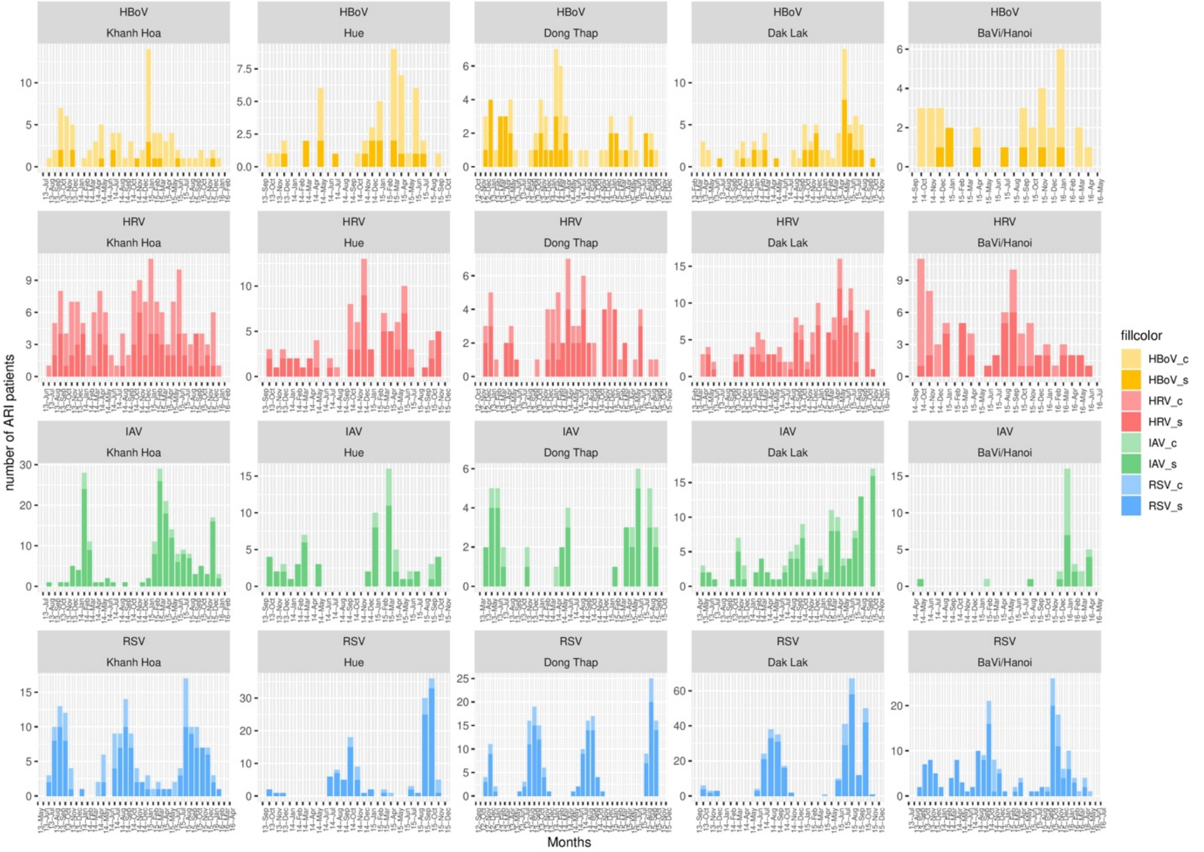

Figure S2 Monthly distribution of the 4 major viruses (RSV, HRV, IAV and HBoV) detected in ARI patients admitted to hospital in different site from October 2012 to June 2016. Colors of different viruses are consistent with Figure 1 and 2, shallower colors are multiple infections (c), darker colors are single infections (s).

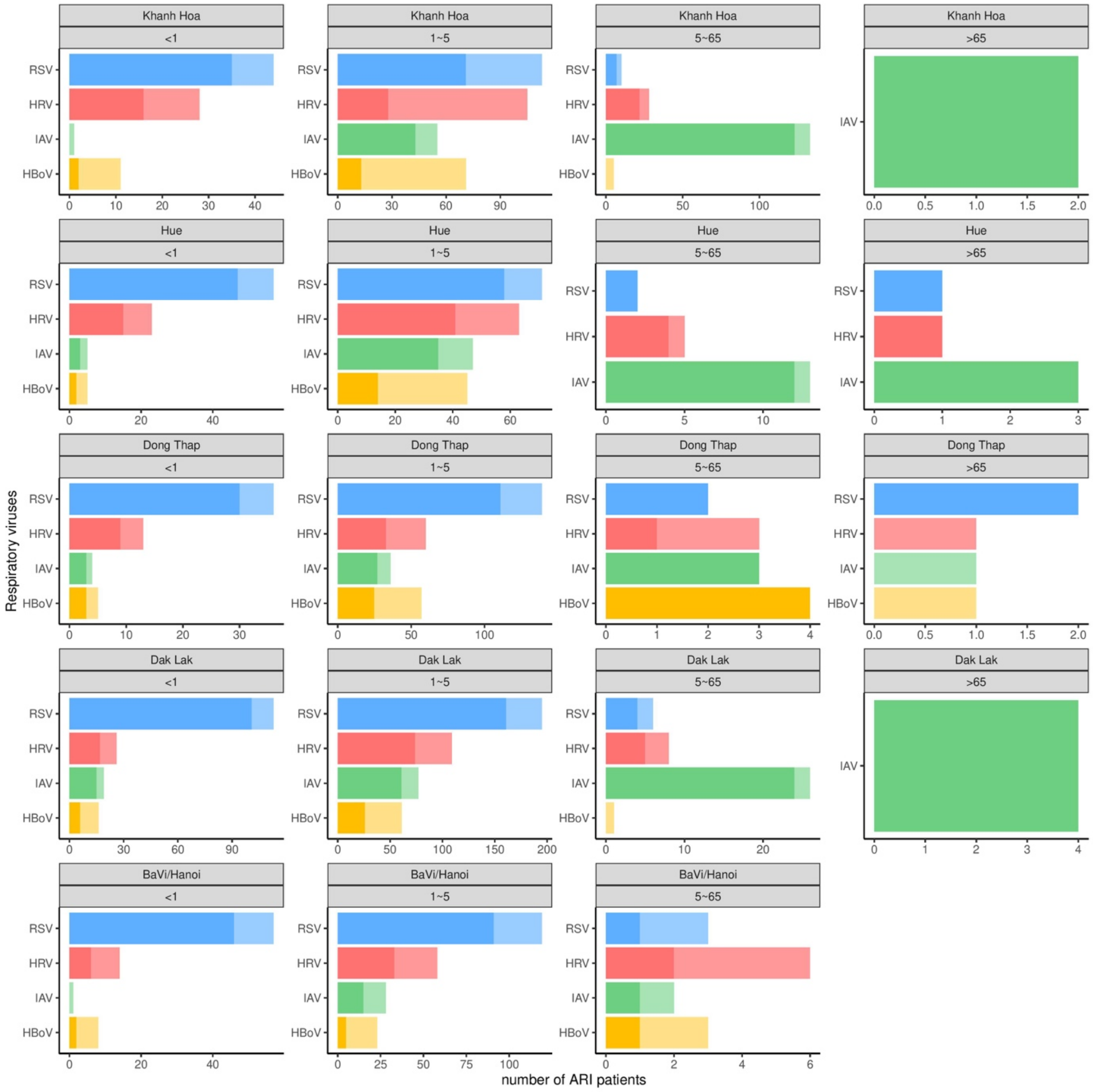

Figure S3 Proportions of virus co-detections among different sites and age groups. The number of ARI patients infected with a single virus (darker shade) and multiple viruses.

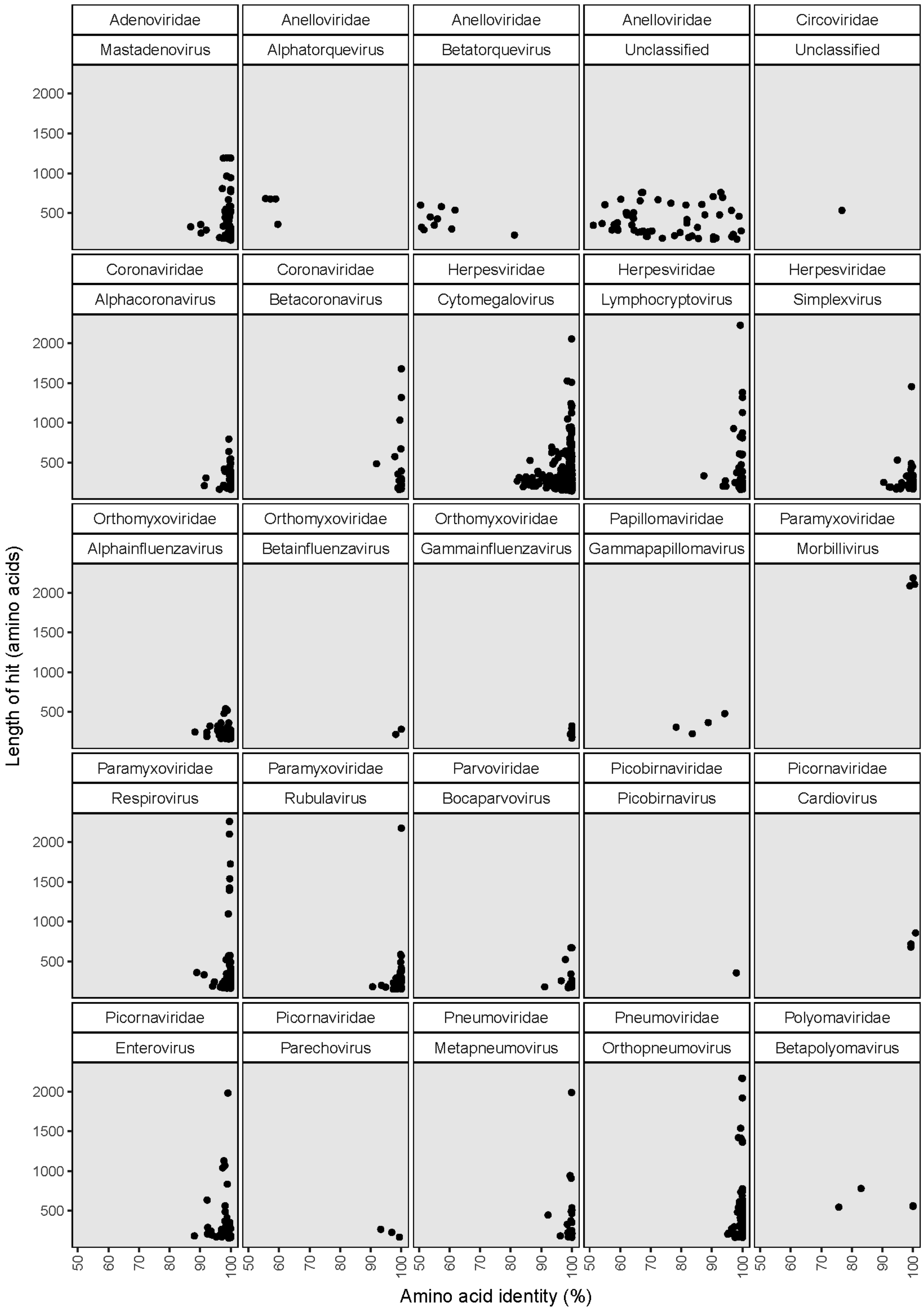

Figure S4 Distribution of virus sequences (translated protein) detected by metagenomic sequencing. Assembled contigs were classified by viral family and genus determined by BLASTX. Only contigs (n=1363) with quality assurance (Contig length >= 500 nt, hit length >= 200aa, score >= 500, amino acid identity >= 50%) were shown.

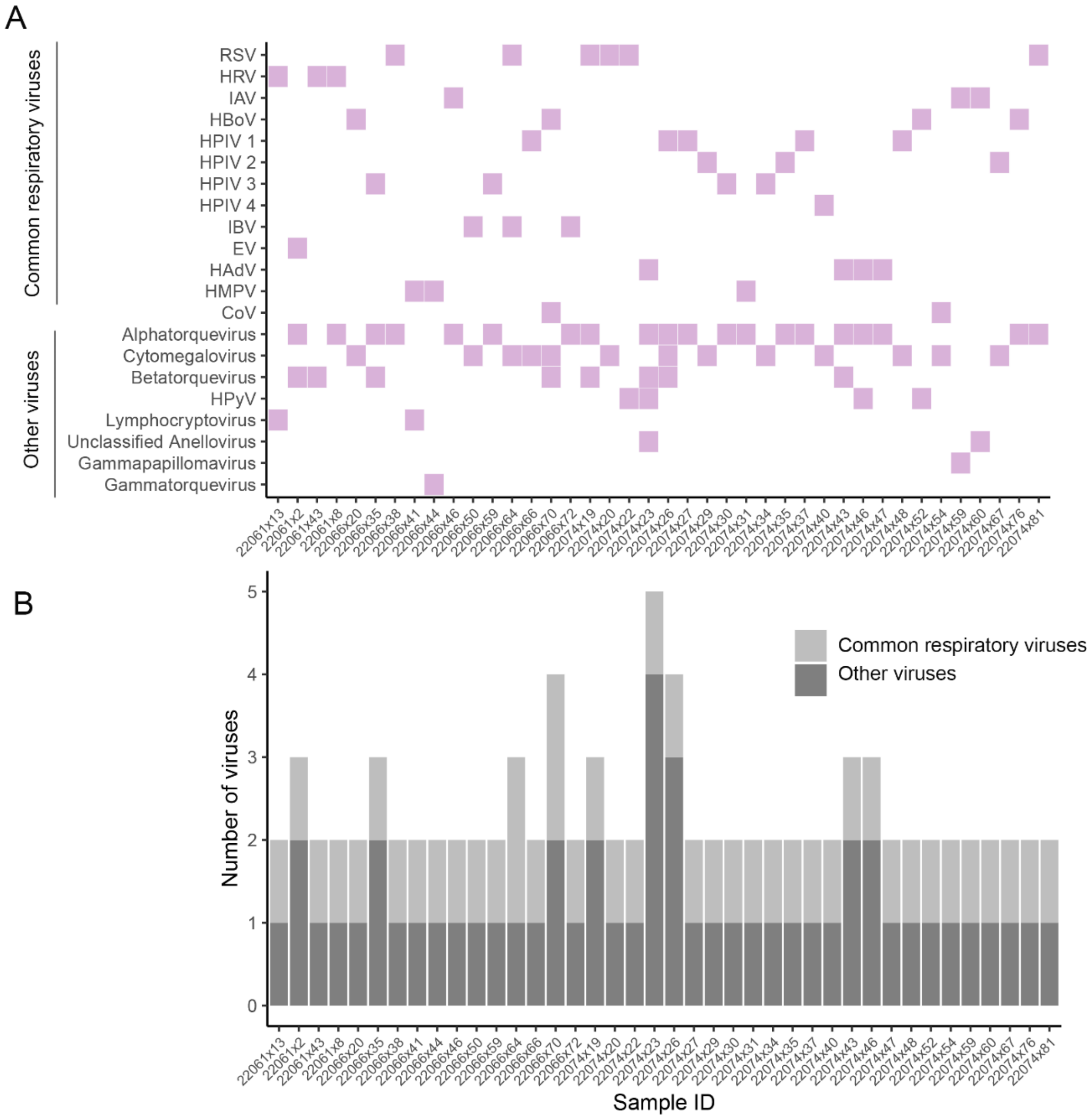

Figure S5 Concurrence of common respiratory viruses and other viruses in same patients (n=40) detected by metagenomics sequencing. (A) Specific viruses found in each patients co-infected and (B) the number of viruses detected in each patients were shown, with common respiratory viruses and other viruses listed separately.

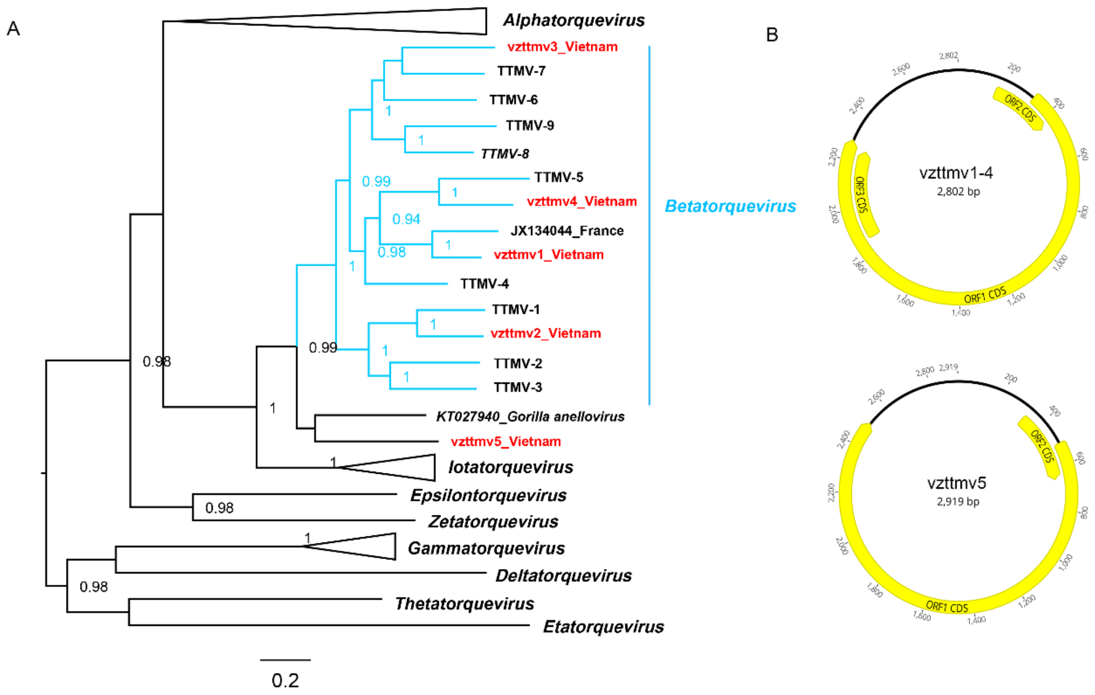

Figure S6 Novel anellovirus genomes found in respiratory patients. A) Maximum likelihood trees of members of *Anelloviridae* family (ORF1 gene sequences). Phylogenetic relations between currently ICTV taxonomy recognized *Anelloviridae* (genus and species) and Vietnam isolates in this study (highlight in red) were shown. Genera with more than 1 species are collapsed. The *Betatorquevirus* genus clade was highlight in blue, with species Torque teno mini virus (TTMV 1-9) and other close related reference sequences labelled on tips. The bootstrap value >0.9 were shown on nodes. B) Predicted genetic organization of anellovirus genomes (vzttmv1-4 and vzttmv5). The name and length of the determined nucleotide sequences of the viral species are shown in the center of the genomic scheme. The putative open reading frames (ORFs) are showed in yellow.

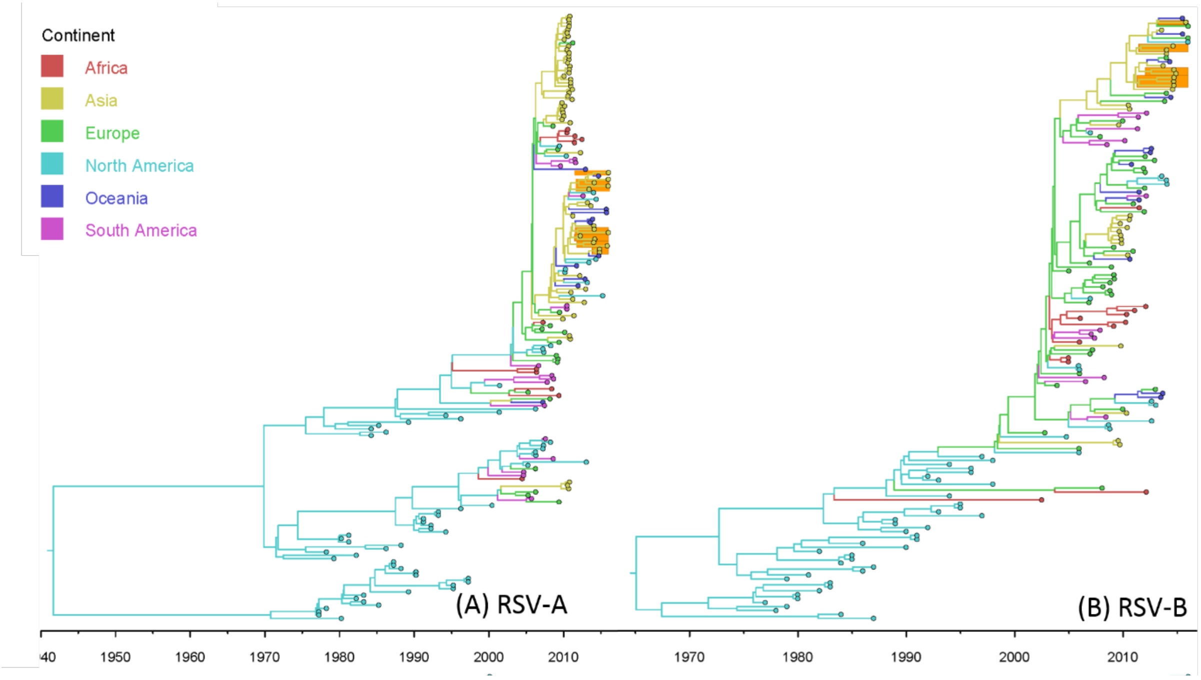

Figure S7 Bayesian maximum clade credibility trees for RSV-A and RSV-B whole genome sequences of RSV A and B in Vietnam and worldwide in (A) RSV-A; (B) RSV-B. Branch colors represent the most probable ancestral locations of each branch, inferred using discrete trait model; sequences found in this study are highlight in orange

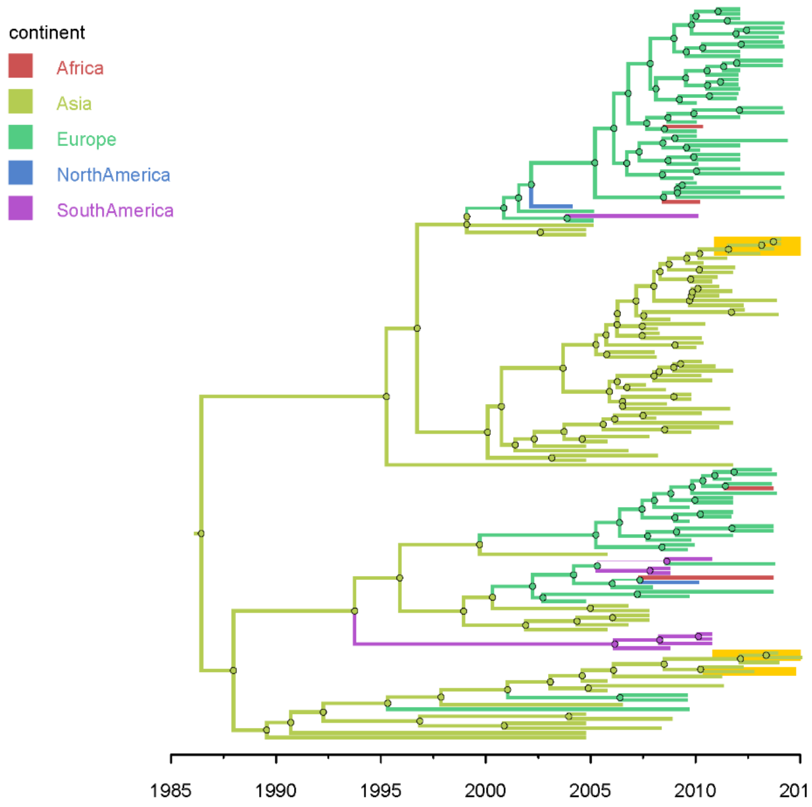

Figure S8 Bayesian MCC trees of Vp1 gene of Bocavirus 1. Branch colors represent the most probable ancestral locations of each branch, inferred using discrete trait model; sequences found in this study are highlight in orange (N=7).

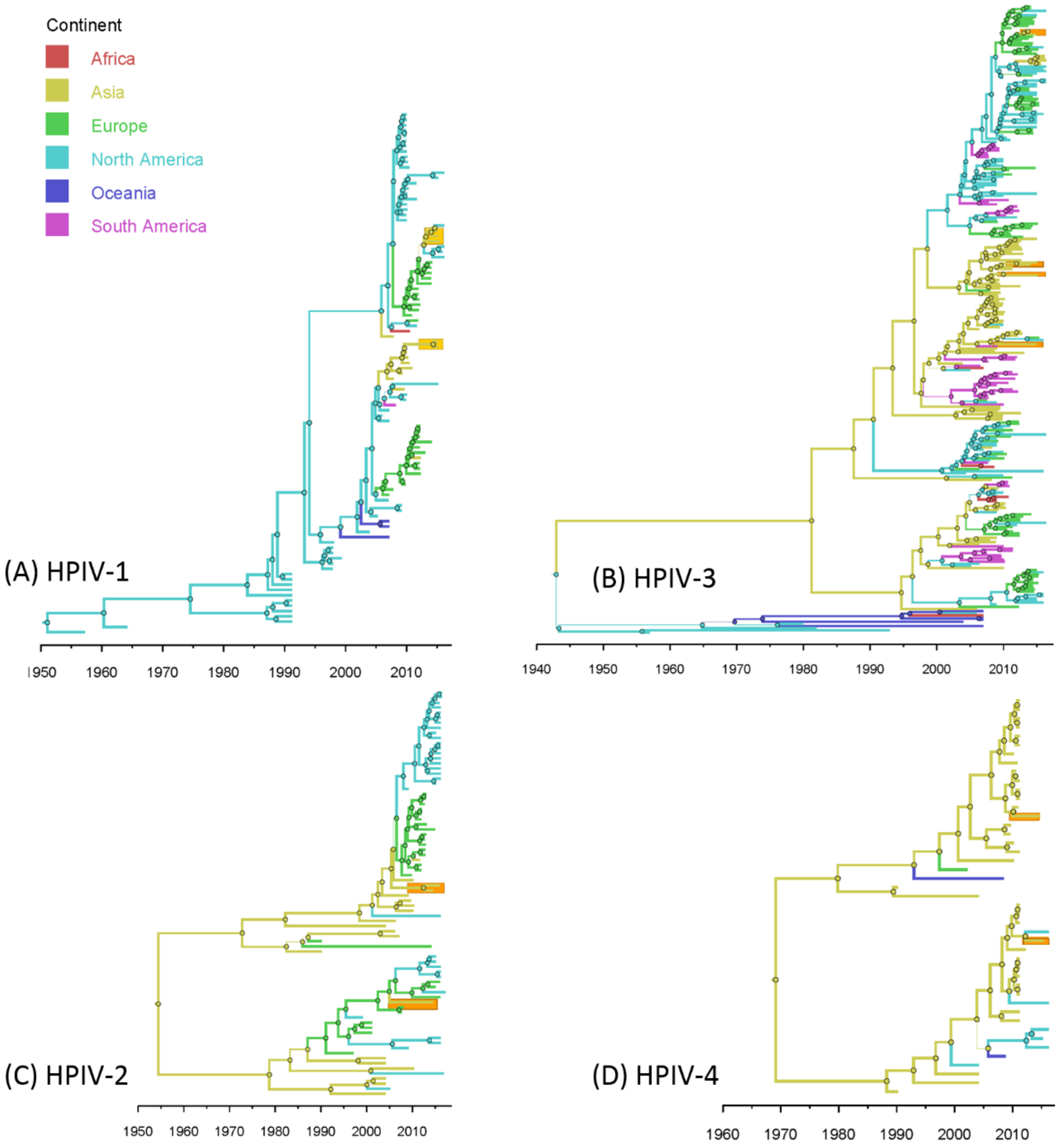

Figure S9 Bayesian MCC trees of HN gene of *Parainfluenza virus* (HPIV) 1-4 in Vietnam and worldwide. (A) Parainfluenza 1 (*Respiratory virus 1*); (B) Parainfluenza 3 (*Respiratory virus 3*); (C) Parainfluenza 2 (*Human rubulavirus 2*) and (D) Parainfluenza 4 (*Human rubulavirus 4*). Branch colors represent the most probable ancestral locations of each branch, inferred using discrete trait model; sequences found in this study are highlight in orange.

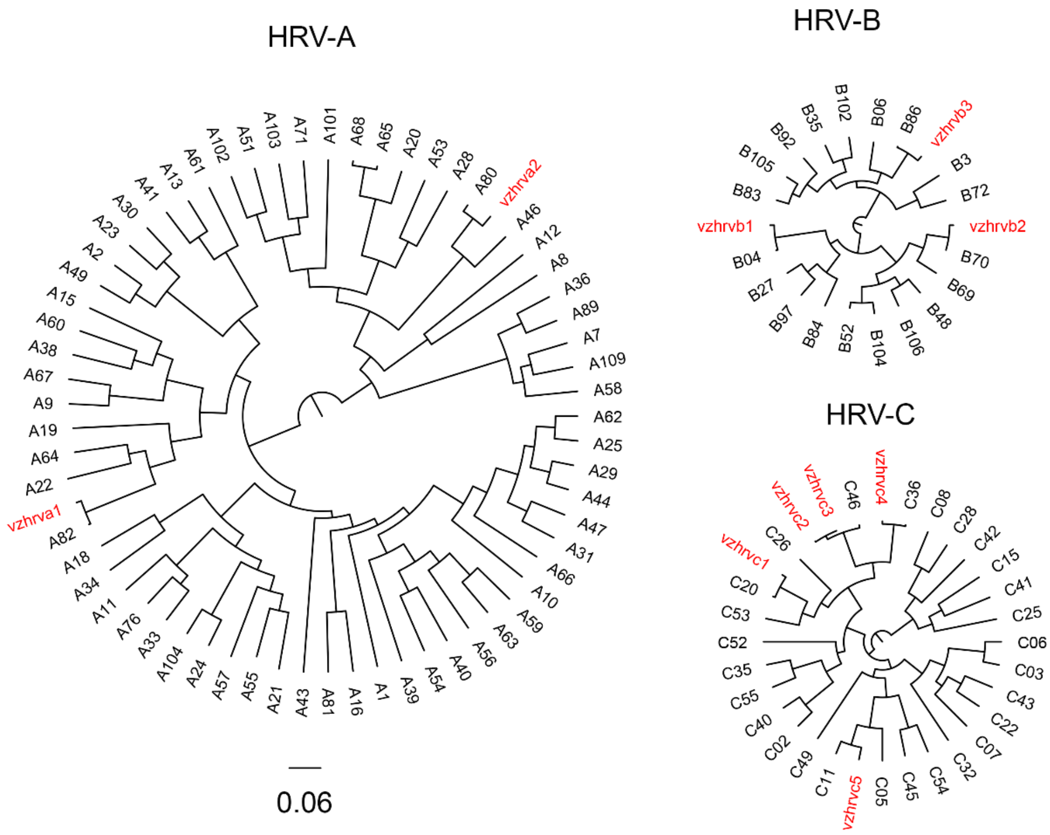

Figure S10 Maximum likelihood trees of Vp1 gene of human rhinovirus (HRV) genotype A, B and C. Phylogenetic relations between currently known HRV serotypes and Vietnam isolates in this study (highlight in red) were shown.

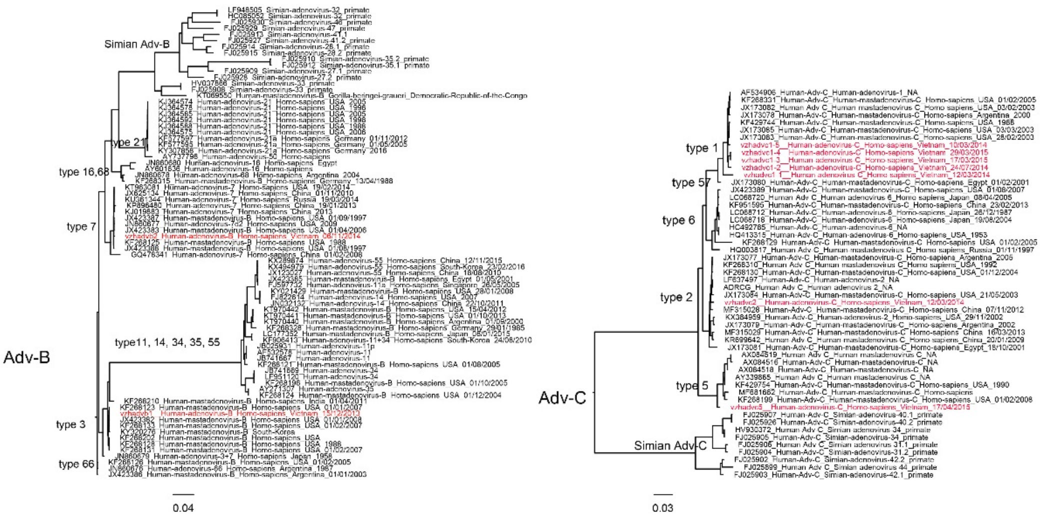

Figure S11 Maximum likelihood trees of whole genome sequences of *Adenovirus* (Adv-B and Adv-C). Sequences found in this study are highlight in red.

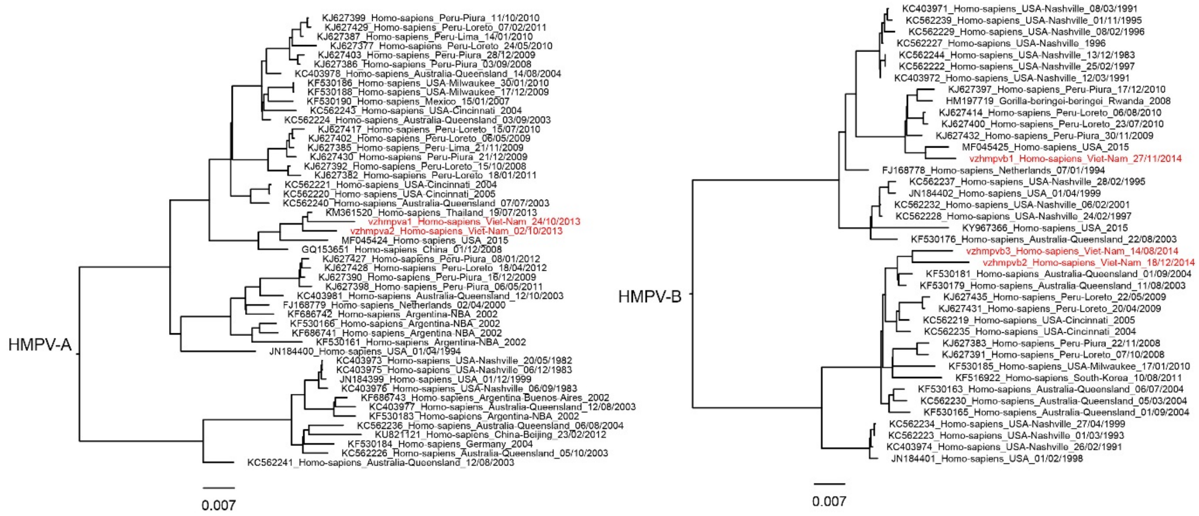

Figure S12 Maximum likelihood trees of full genomes of *Human metapneumovirus* (HMPV) genotype A and B. Sequences found in this study are highlight in red.

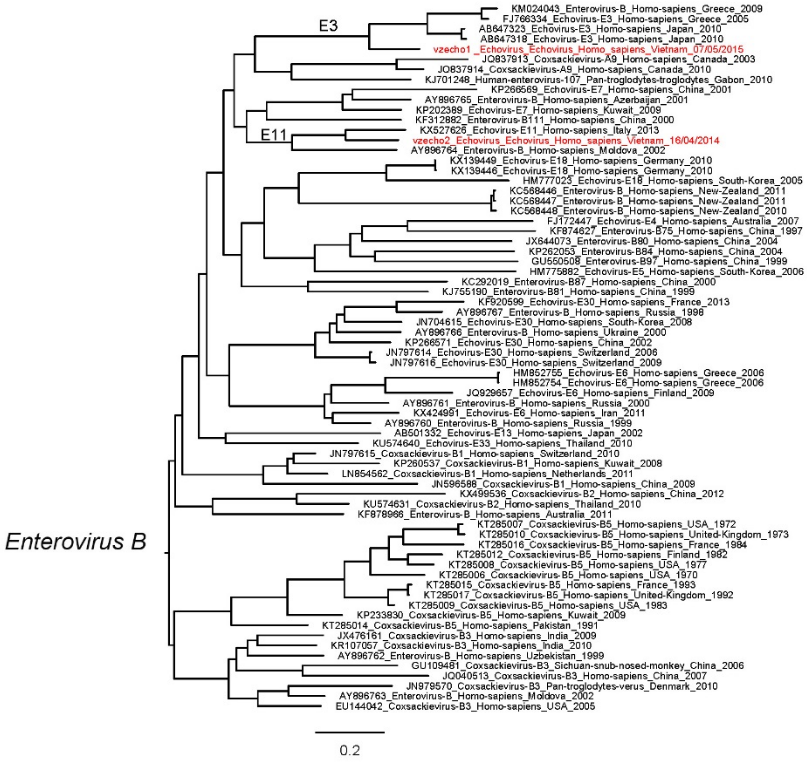

Figure S13 Maximum likelihood trees of full genomes of *Enterovirus B*. Sequences found in this study are highlight in red.

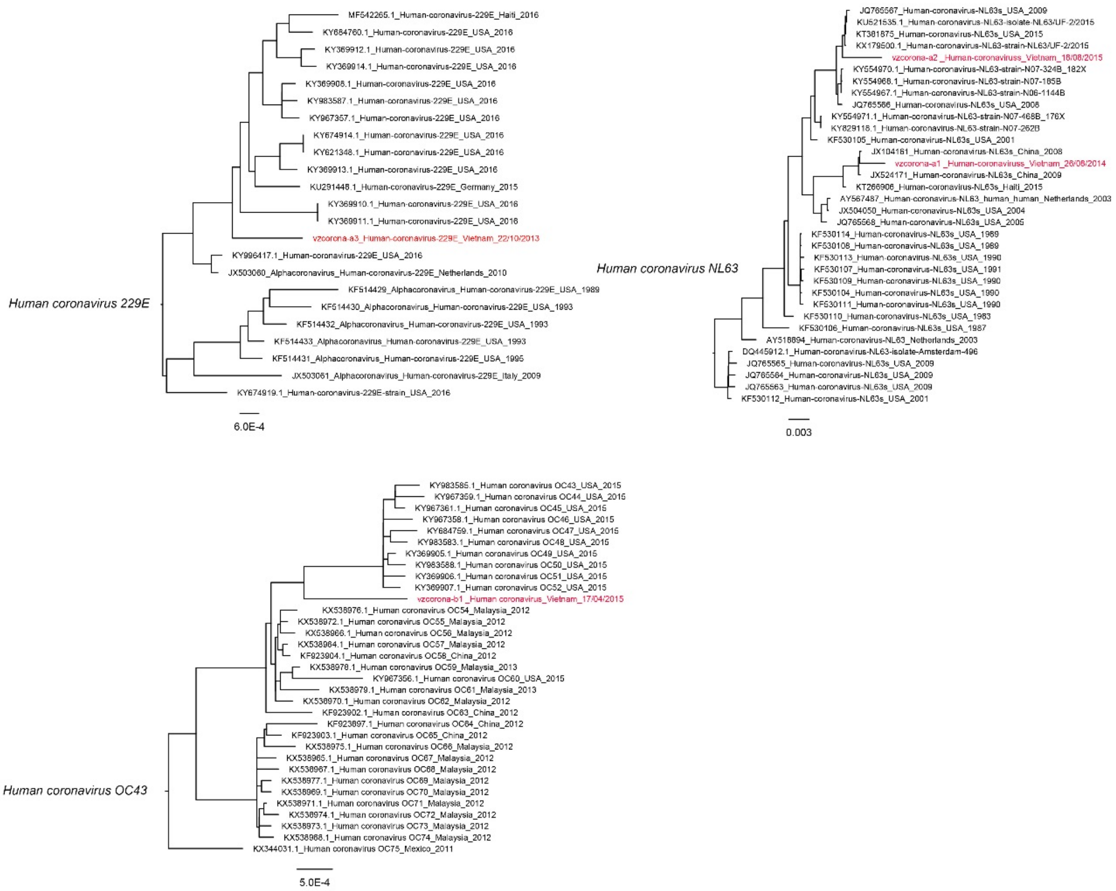

Figure S14 Maximum likelihood trees of full genomes of *Coronaviridae (Human coronavirus 229E, Human coronavirus NL63 and Human coronavirus OC43)*. Sequences found in this study are highlight in red.

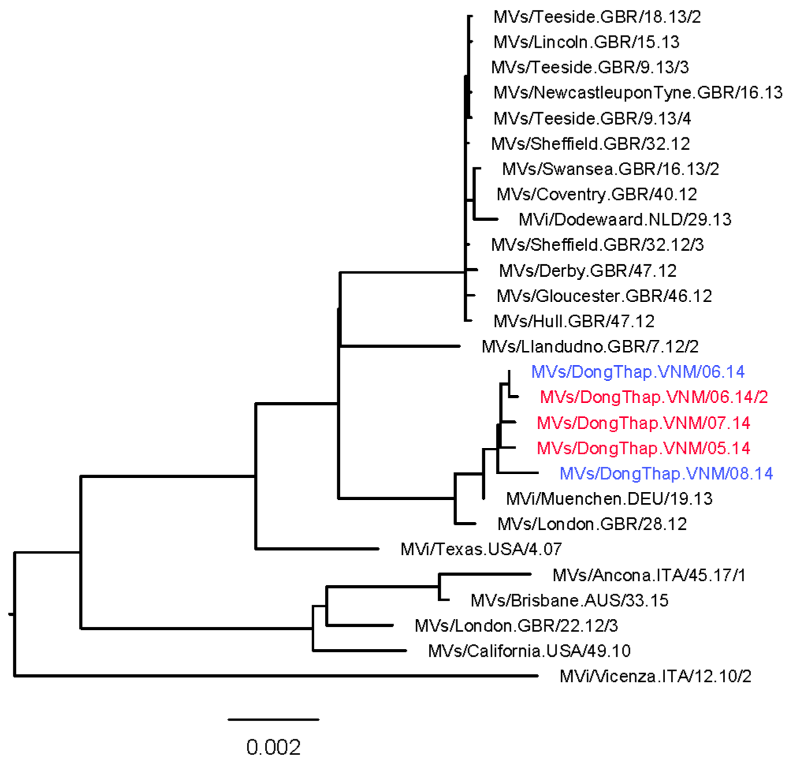

Figure S15 Maximum likelihood trees of whole genome sequences of *Measles morbillivirus* genotype D8. Sequences found in this study are highlight in red; Sequences isolated from enteric patients in the equivalent surveillance are highlight in blue

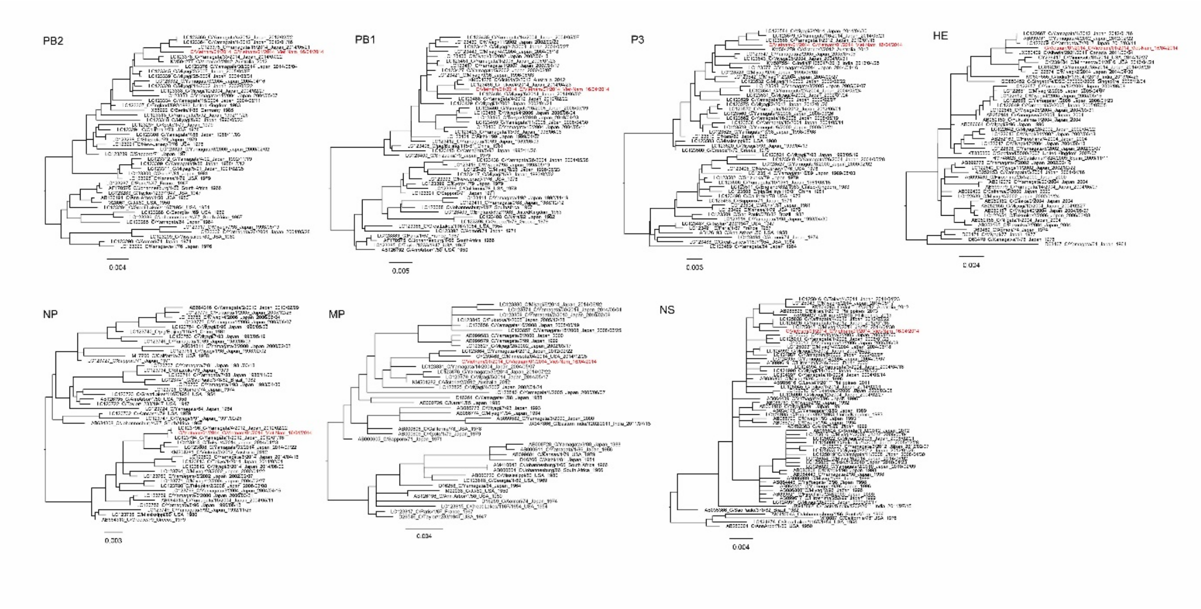

Figure S16 Maximum likelihood trees of all gene segments (n=7) of *Influenza C virus*. Sequences found in this study are highlight in red.

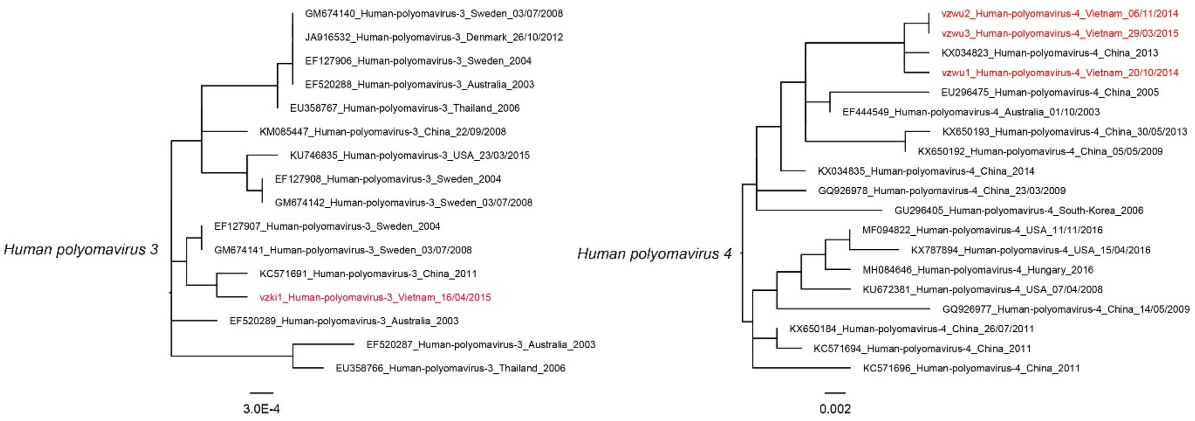

Figure S17 Maximum likelihood trees of whole genome sequences of *Human polyomavirus 3 and Human polyomavirus 4*. Sequences found in this study are highlight in red.
